# Systematic Review and Meta-analysis of the Predictive Performance of Stroke and Bleeding Prediction Models in Atrial Fibrillation Patients With Kidney Disease

**DOI:** 10.1016/j.xkme.2025.101200

**Published:** 2025-12-11

**Authors:** Liselotte F.S. Langenhuijsen, Daniëlle C.L. Derksen, Jet Milders, Sabine F.B. van der Horst, Merel van Diepen, Serge A. Trines, Paul L. den Exter, Frederikus A. Klok, Joris I. Rotmans, Ype de Jong

**Affiliations:** 1 Department of Clinical Epidemiology, Leiden University Medical Center, Leiden, The Netherlands; 2 Department of Thrombosis and Haemostasis, Leiden University Medical Center, Leiden, The Netherlands; 3 Department of Internal Medicine, Noordwest Ziekenhuisgroep, Alkmaar, The Netherlands; 4 Department of Cardiology, Leiden University Medical Center, Leiden, The Netherlands; 5 Department of Internal Medicine (Nephrology), Leiden University Medical Center, Leiden, The Netherlands

**Keywords:** Prediction, stroke, bleeding, atrial fibrillation, kidney disease, renal failure, dialysis, bias

## Abstract

**Rationale & Objective:**

Patients with atrial fibrillation (AF) and chronic kidney disease (CKD) are at high risk for ischemic stroke (IS) and bleeding. The applicability of prediction models in this population remains debated. This study aimed to (1) identify external validations of CHA_2_DS_2_-VASc, CHADS_2_, HAS-BLED, and HEMORR_2_HAGES model scores in patients with AF undergoing dialysis or with CKD, (2) provide pooled estimates, and (3) assess their risk of bias (ROB).

**Study Design:**

Systematic review and meta-analysis.

**Setting & Participants:**

We searched Web of Science, PubMed, MEDLINE, Embase, Emcare, PMC, Cochrane Library, and Academic Search Premier for studies externally validating IS and bleeding prediction models in patients with AF undergoing dialysis or with CKD.

**Exposures:**

AF and CKD or dialysis.

**Outcomes:**

IS and bleeding.

**Analytical Approach:**

Eligible studies were reviewed, discrimination was pooled using random-effects meta-analysis, calibration was calculated and plotted, and the ROB score was assessed using the prediction model ROB assessment tool.

**Results:**

The CHA_2_DS_2_-VASc score was validated in 35 studies, the CHADS_2_ and HAS-BLED scores in 19 each, and the HEMORR_2_HAGES score in 1. Among 627,199 patients, 28,493 (4.5%) experienced IS and 25,695 (4.1%) bleeding. Only 12 studies presented c-statistic scores. In patients with AF and CKD, the CHADS_2_ model score showed nominally better discrimination predicting IS (pooled c-statistic score of 0.70) than the CHA_2_DS_2_-VASc model score (0.64). In patients with AF undergoing dialysis, the CHA_2_DS_2_-VASc and CHADS_2_ model scores showed similar discrimination predicting IS (both 0.70), and the HAS-BLED and HEMORR_2_HAGES model scores showed similar c-statistic scores predicting bleeding (0.55 and 0.56, respectively). Calibration was good in the most relevant high-risk group.

**Limitations:**

All studies were at high ROB scores, contained within- and between-study heterogeneity, and often merged scoring categories or populations, limiting comparability.

**Conclusions:**

Although modest, the discrimination of prediction models in patients with AF undergoing dialysis or with CKD is similar to patients with AF without CKD. Despite the described limitations, these models can be used in clinical practice for patients with CKD and patients undergoing dialysis.

## Plain-Language Summary

Patients with both atrial fibrillation (AF) and chronic kidney disease (CKD) or patients undergoing dialysis face a higher risk of stroke and therapy-related bleeding. To estimate these risks, prediction models can be used, but their predictive value in this population is unclear. This study reviewed and analyzed existing studies on 4 commonly used stroke and bleeding prediction models in patients with AF undergoing dialysis or with CKD. We found that, although these models showed modest discrimination, their performance was similar to that in patients with AF without CKD or undergoing dialysis. Therefore, despite the weaknesses of the included studies, we believe these tools can be used for patients with CKD and patients undergoing dialysis to help guide treatment decisions.

Key findings:

• This study identified 35 studies validating the CHA_2_DS_2_-VASc score, 21 the CHADS_2_ score, 22 the HAS-BLED score, and 1 the HEMORR_2_HAGES score, reflecting guideline endorsement.
• In atrial fibrillation (AF) patients with chronic kidney disease (CKD), the CHADS_2_ score shows nominally better discriminative abilities than the CHA_2_DS_2_-VASc score.
• In patients undergoing dialysis, the CHA_2_DS_2_-VASc, CHADS_2_, HAS-BLED, and HEMORR_2_HAGES scores perform similarly.
• The CHA_2_DS_2_-VASc and HAS-BLED scores showed good calibration in the most relevant high-risk group.
• High risk of bias (ROB) was found across studies, especially in the Outcome and Analysis domains.

What this adds to what was known:

• Though modest, the discriminative abilities of these models in patients with AF undergoing dialysis or with CKD are comparable with those in patients with AF with normal kidney function.
• Calibration was poor in lower-risk groups but showed better agreement in the more relevant higher-risk groups.
• The ROB in these prediction model studies is high.

What is the implication, and what should change now?

• Aligning with broader AF guidelines, conventional stroke and bleeding prediction models can also be applied to patients with CKD and patients undergoing dialysis.
• The high ROB scores of the included studies and heterogeneity of the included populations highlight the need for more rigorous validations of prediction models in this population.

## Graphical abstract

**Figure:**
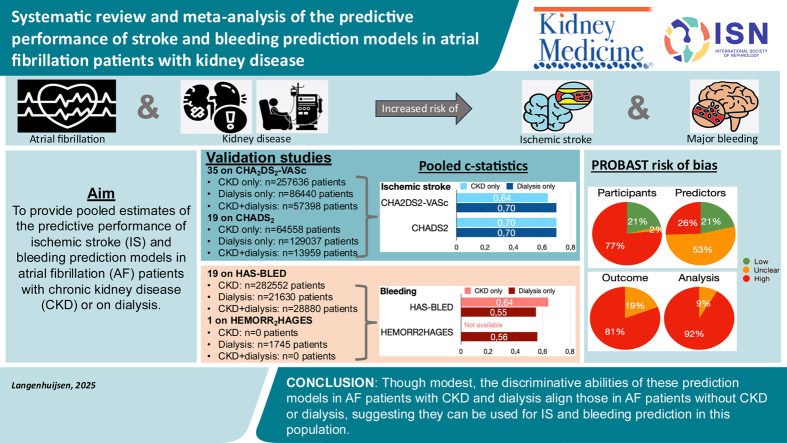

The benefit of anticoagulation in patients with atrial fibrillation (AF) undergoing dialysis or with chronic kidney disease (CKD) is a debated topic because patients with decreased kidney function are at high risk for both ischemic stroke (IS) and therapy-related bleeding.^1^, ^2^, ^3^ This can be explained by a combination of CKD-specific risk factors (eg, accelerated atherosclerotic vascular disease) and general risk factors (eg, aging, hypertension).^4^, ^5^, ^6^ Multiple prediction models have been developed to predict the risk of IS or bleeding. The European Society of Cardiology (ESC) and Kidney Disease Improving Global Outcomes (KDIGO) guideline advise weighing these risks in patients with AF, using the CHA_2_DS_2_-VASc model (or the CHA_2_DS_2_-VA model [ie, excluding sex] or, in previous guidelines, the CHADS_2_ model) for IS and the HAS-BLED model for bleeding risk, before intervening on modifiable risk factors (eg, hypertension) or starting anticoagulation therapy.^7^^,^^8^ Although the KDIGO guideline offers a more detailed approach per CKD stage, both guidelines advise starting anticoagulation therapy if indicated by a positive CHA_2_DS_2_-VASc score (thresholds differ between the ESC and KDIGO guidelines), thereby weighing the risks of IS and bleeding. Another frequently used model to predict bleeding risk is the HEMORR_2_HAGES model.^9^^,^^10^ Of these models, only the HAS-BLED model included a predictor for kidney function in their model. The other models do not mention the kidney function in their derivation cohorts.

Though these 4 commonly used prediction models have been validated in general populations with AF, with development and pooled external validations c-statistic scores of 0.61 and 0.64, respectively, for the CHA_2_DS_2_-VASc model; 0.82 and 0.66 for the CHADS_2_ model; 0.72 and 0.65 for the HAS-BLED model; and 0.67 and 0.63 for the HEMORR_2_HAGES model,^10^, ^11^, ^12^, ^13^, ^14^, ^15^ their applicability to patients with AF undergoing dialysis or with CKD remains questionable because of the conflicting results of validation studies in this population.^5^^,^^16^ This notion is underlined by the limited attention for CKD in the ESC guideline on AF, which describes uncertainty regarding the use of oral anticoagulants (OACs) to prevent IS in this population.^7^ Furthermore, a negative relation between kidney function and model performance has been demonstrated before, showing a decline in discrimination of most IS-models in CKD stage 3 and stage 4-5 compared with stage 1-2.^5^ This uncertainty was illustrated by a recent study showing heterogeneity in physicians’ initiation of OAC therapy and which type of OAC they start.^17^ With guidelines leaving room for clinical judgment and validation studies showing conflicting results regarding the robustness of prediction models, clinicians need guidance on how to facilitate individualized IS and bleeding risk assessments for their patients.^5^^,^^6^^,^^18^^,^^19^

Therefore, the aims of this systematic review and meta-analysis were to (1) identify external validations of the CHA_2_DS_2_-VASc, CHADS_2_, HAS-BLED, and HEMORR_2_HAGES models for predicting IS or bleeding in patients with AF undergoing dialysis or with CKD, (2) provide pooled estimates of the predictive performance of these models, and (3) assess their risk of bias (ROB) scores.

## Methods

The current review is reported following the Preferred Reporting Items for Systematic reviews and Meta-Analyses (PRISMA) guideline, the Transparent Reporting of a multivariable prediction model for Individual Prognosis Or Diagnosis (TRIPOD) statement, and the CHecklist for critical Appraisal and data extraction for systematic Reviews of prediction Modelling Studies (CHARMS) guideline.^20^, ^21^, ^22^ Because this systematic review and meta-analysis used previously published data and did not involve any personally identifiable information, this study was exempt from formal ethics committee approval and did not require informed consent.

### Search Methods and Eligibility

Two searches were conducted to identify studies using the CHA_2_DS_2_-VASc, CHADS_2_, HAS-BLED, and HEMORR_2_HAGES models for IS and bleeding prediction in CKD and dialysis patients with AF. Searches were drafted by an experienced medical librarian and based on previous searches conducted by our team in similar settings.^5^^,^^15^^,^^23^, ^24^, ^25^ We performed (1) a search in Web of Science to identify all studies citing any of these 4 prediction models and (2) a search in PubMed, MEDLINE, Embase, Emcare, PMC, Cochrane Library, and Academic Search Premier, using these prediction models as a search term (see Item S1 for the detailed search method). These searches were conducted on the 11th of June 2021 and updated on the 30th of January 2024. We then filtered the combined dataset using search terms specific to CKD and dialysis and search terms relevant for validation studies, as done before.^15^ Studies were excluded when they (1) contained a mixed population of CKD and non-CKD or AF and non-AF without providing information specific to our population of interest, (2) omitted information on the predictive performance (discrimination or calibration, or relevant data that would allow us to calculate a measure of predictive performance), or (3) did not include IS or bleeding as an outcome.

### Data Extraction and Study Appraisal

Titles, abstracts, and full texts were independently screened by 2 researchers (DCLD/LFSL). Conflicts were discussed with a third reviewer (YdJ). Data extraction was conducted by DCLD and LFSL using a predefined data extraction sheet (see Item S2) and checked for accuracy by LFSL and YdJ, respectively. Demographic data of the included cohorts (including age, sex, ethnicity, and country of origin) were extracted. For studies including patients with CKD, the stage of CKD (KDIGO stages I-V) was extracted where available. For studies including patients undergoing dialysis, the type of dialysis (hemodialysis [HD] or peritoneal dialysis [PD]) was extracted. AF cohorts were labeled as incident (ie, patients with newly developed AF), prevalent (ie, patients with known AF), or unclear type of AF. OAC use and type of OAC were extracted. Outcome definitions (ie, IS for the CHA_2_DS_2_-VASc and CHADS_2_ models and bleeding for the HAS-BLED and HEMORR_2_HAGES models) and methodological data, including the type of cohort, were extracted. We also extracted the prediction window (ie, the time between prediction at baseline and the timeframe in which the outcome may occur). To compare the discrimination of prediction models, describing the extent to which a model predicts a higher risk for patients with the outcome compared with those without the outcome, all available c-statistic scores were extracted.^26^ Calibration data were extracted, and if formal calibration methods were omitted, incidence rates (event rates) or cumulative incidences stratified per risk score stratum (eg, observed risk per CHA_2_DS_2_-VASc point) were extracted. To allow comparison, describing the accuracy with which the predicted risk corresponds to the observed risk, we approximated the cumulative incidence for studies presenting event rates as done before.^5^^,^^23^ Methodological quality was independently assessed and cross-checked by 2 reviewers (DCLD/LFSL) using the Prediction model Risk Of Bias ASsessment Tool (PROBAST), a ROB tool consisting of 20 signaling questions structured in 4 domains (Participants, Predictors, Outcome, and Analysis).^27^ Conflicts in scoring were discussed until consensus was reached.

### Statistical Analysis

To assess the level of agreement between our researchers in the study selection process, we calculated a Cohen’s Kappa. Model discrimination was assessed using the c-statistic score, also known as the area under the receiver curve. A c-statistic score of 0.50 reflects pure chance; 0.50-0.59 is regarded as poor, 0.60-0.75 as modest, and above 0.75 as good discrimination. We conducted a random-effects meta-analysis to summarize c-statistic scores. C-statistic scores were logit-transformed, and confidence intervals (CIs) were calculated using the Hartung-Knapp-Sidik-Jonkman approach. This approach is used to calculate CIs of estimated effect sizes in small-sized meta-analyses by accounting for the heterogeneity of effect sizes across studies and adjusting the standard error of the estimate.^28^, ^29^, ^30^ These logit pooled c-statistic scores were calculated for all included models and then transformed back to the original scale. Forest plots were drawn to visualize the estimated results of all included studies. Statistical heterogeneity was quantified using the I^2^ statistic. To evaluate the potential small-study effect, the c-statistic score and its standard error were used to create funnel plots. Funnel plot asymmetry was evaluated using Egger’s test for analyses including ≥10 studies, with *P* values ≤0.05 indicating a statistically significant small-study effect. Calibration plots were drafted to compare the observed risks per score stratum in the validation studies with the observed risks in the development studies (ie, the predicted risks), as done before.^31^ As most studies aggregated observed risks for multiple strata (eg, for CHA_2_DS_2_-VASc scores of 0-3), we calculated an unweighted average predicted risk for these aggregated strata and compared this to the aggregated observed risk. We refrained from pooling the agreement between observed and predicted risk because the aggregated risk strata differed per study but provided an unweighted average of the calibration-in-the-large per stratum for all scores. All analyses were conducted using RStudio v4.1.2 and the metafor and forest plot packages.^32^

### Sensitivity Analyses

For the main analyses, we explored the predictive performance of prediction models in patients with AF undergoing dialysis and with CKD, including studies with unclear baseline characteristics (eg, mixed populations of CKD and dialysis patients and mixed populations of incident, prevalent, or unclear type AF). We also included studies with different study designs (ie, all studies presenting c-statistic scores were prospective and retrospective (cohort) studies; no randomized controlled trials were included). Because indication bias may confound observations, we conducted 6 sensitivity analyses to study the effect of the inclusion of studies with unclear baseline patient characteristics and studies with different study designs. Sensitivity analyses were performed as our main analysis but included:

(1) Only patients with prevalent CKD (all stages) and dialysis;
(2) Only patients with unclearly defined CKD and dialysis (eg, probable mixed groups of CKD, dialysis, or patients receiving kidney transplants);
(3) Only patients with incident AF;
(4) Only patients with prevalent and unclear type AF;
(5) Only studies with a prospective study design;
(6) Only studies with a retrospective study design.

## Results

### Systematic Search and Study Characteristics

**Figure 1:**
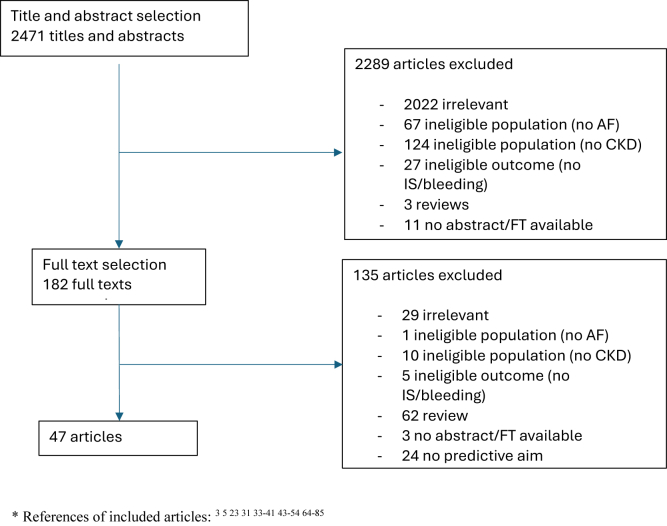
Flow chart for study selection. ∗ References of included articles:^3^^,^^5^^,^^23^^,^^31^^,^^33^, ^34^, ^35^, ^36^, ^37^, ^38^, ^39^, ^40^, ^41^^,^^43^, ^44^, ^45^, ^46^, ^47^, ^48^, ^49^, ^50^, ^51^, ^52^, ^53^, ^54^^,^^64^, ^65^, ^66^, ^67^, ^68^, ^69^, ^70^, ^71^, ^72^, ^73^, ^74^, ^75^, ^76^, ^77^, ^78^, ^79^, ^80^, ^81^, ^82^, ^83^, ^84^, ^85^

After removal of duplicates, the searches yielded 2,471 results, of which 47 studies met the eligibility criteria (Fig 1 contains the study selection process, and Table S1 provides an overview of the included studies). The independent study selection showed good agreement, with a Cohen’s Kappa of 0.90 for the combined title-abstract selection and 1.00 for the full-text selection. Of these 47 studies, 35 assessed the CHA_2_DS_2_-VASc model (12 including patients with CKD, 17 including patients undergoing dialysis, and 6 including both patients with CKD and patients undergoing dialysis), 19 assessed the CHADS_2_ model (7 on patients with CKD, 10 on patients undergoing dialysis, and 2 including both), 19 assessed the HAS-BLED model (8 on patients with CKD, 9 on patients undergoing dialysis, and 2 including both), and 1 assessed the HEMORR_2_HAGES model (including patients undergoing dialysis). In total, 11 studies were conducted in an incident AF cohort, 26 in a prevalent AF cohort, 7 in an unclear type AF cohort, and 3 in a combined incident and prevalent AF cohort. The included studies consisted of 16 studies on patients with CKD (including 365,715 patients), 25 studies on patients undergoing dialysis (including 204,086 patients), and 6 studies on both patients with CKD and patients undergoing dialysis (including 57,398 patients) (Table S2). In total, these studies contained 627,199 patients, of whom 28,493 experienced IS and 25,695 experienced bleeding, with reported IS occurrence rates of 0.5%-40.1% and bleeding rates of 0.7%-67.0%. When specified, HD was more prevalent than PD. Ten studies were conducted in a CKD population where all stages (1-5) were represented. 2 studies covered CKD stages 1-4, 2 stages 1-3, 2 stages 3-5, 1 stage 4-5, and 1 stage 5. Outcome definitions of studies validating the CHADS_2_ and CHA_2_DS_2_-VASc models were largely similar. Bleeding definitions varied, with some studies including a composite of major and minor bleeding. A total of 21 studies did not provide an outcome definition. Sample sizes ranged from 60-65,734 participants, with median ages ranging from 52.2-78.4 years. Most studies (n=15) were retrospective cohort studies. Three studies conducted a randomized controlled trial, 2 of which were blinded.

## Systematic Review

### Discrimination

C-statistic scores were presented in 12 studies.^5^^,^^23^^,^^31^^,^^33^, ^34^, ^35^, ^36^, ^37^, ^38^, ^39^, ^40^, ^41^ In patients with CKD, c-statistic scores were stratified per CKD stage or aggregated for multiple stages (eg, stages 1-3 and 4-5). Table 1 provides an overview of the groups of stages per validation study and the corresponding c-statistic scores for patients with CKD. Table 2 provides an overview of the c-statistic scores per dialysis modality (ie, HD or HD and PD combined).

**Table 1:** Discrimination of the CHADS_2,_ CHA_2_DS_2_-VASc, and HAS-BLED Model Scores in Patients With CKD

		Study	CKD Stage				
			1	2	3	4	5
Ischemic stroke	CHA_2_DS_2_-VASc model score	Apostolakis et al^37^	0.67 (0.60-0.75)				
		Roldan et al^40^	0.623 (0.592-0.654)				
		Friberg et al^39^^a^	0.71 (0.71-0.72)				
		Bautista et al^36^^b^	0.602 (0.55-0.65)				
		Bautista et al^36^^b^	0.579 (0.52-0.64)			0.623 (0.51-0.73)	
		De Jong et al^5^	0.70 (0.69-0.71)		0.60 (0.58-0.62)	0.58 (0.52-0.64)	
	CHADS_2_ model score	Apostolakis et al^37^	0.64 (0.56-0.73)				
		Roldan et al^40^	0.650 (0.619-0.680)				
		Friberg et al^39^^a^	0.72 (0.72-0.73)				
		Bautista et al^36^^b^	0.605 (0.56-0.65)				
		Bautista et al^36^^b^	0.584 (0.53-0.64)			0.629 (0.52-0.74)	
		De Jong et al^5^	0.78 (0.77-0.80)		0.70 (0.68-0.72)	0.71 (0.66-0.76)	
Bleeding	HAS-BLED model score	Suzuki et al^38^	0.64 (0.55-0.72)				

The HEMORR_2_HAGES model score is not presented in Table 1 because none of these studies included information on the discrimination of this prediction model.

CKD, chronic kidney disease.

a Study contains a mixed population with CKD, undergoing dialysis (hemodialysis and peritoneal dialysis), and undergoing renal transplantation.

b Study contains a mixed population of CKD and hemodialysis patients. Both studies are included in this table for the sake of completeness, but are excluded from all “CKD only” and “dialysis only” analyses. They are therefore only included in the relevant “CKD and dialysis combined” analyses.

**Table 2:** Discrimination of the CHADS_2,_ CHA_2_DS_2_-VASc, HAS-BLED, and HEMORR_2_HAGES Models in Patients Receiving Dialysis

	Study	Ischemic Stroke		Bleeding	
		CHA_2_DS_2_-VASc Model Score	CHADS_2_ Model Score	HAS-BLED Model Score	HEMORR_2_HAGES Model Score
HD and PD					
	Chao et al^35^	0.682 (0.673-0.691)	0.608 (0.598-0.617)	-	-
	Friberg et al^39^^a^	0.71 (0.71-0.72)	0.72 (0.72-0.73)	-	-
	Wang et al^33^	0.847 (0.768-0.926)	0.880 (0.797-0.964)	0.498 (0.393-0.603)	-
	Ocak et al^31^ – within 1 y^c^	-	-	0.64 (0.59-0.69)	0.61 (0.55-0.66)
	Ocak et al^31^ – within 3 y^c^	-	-	0.58 (0.54-0.62)	0.56 (0.52-0.61)
	De Jong et al^23^	0.65 (0.57-0.73)	0.61 (0.56-0.66)	-	-
HD					
	Chan et al^34^	-	0.71 (0.61-0.81)	-	-
	Bautista et al^36^^b^	0.602 (0.55-0.65)	0.605 (0.56-0.65)	-	-
	Bel-Ange et al^41^	0.63 (0.54-0.72)	-	0.47 (0.35-0.57)	-

CKD, chronic kidney disease; HD, hemodialysis; PD, peritoneal dialysis.

a Study contains a mixed population with CKD undergoing dialysis (hemodialysis and peritoneal dialysis) and receiving renal transplantation.

b Study contains a mixed population of CKD and hemodialysis patients. Both studies are included in Table 2 for the sake of completeness, but are excluded from all “CKD only” and “dialysis only” analyses. They are therefore only included in the relevant “CKD and dialysis combined” analyses.

c Study included c-statistic scores for the prediction window of <1 year and <3 years. Both c-statistic scores are included in Table 2 for the sake of completeness, but in our analyses, only the <1 year c-statistic score of the HAS-BLED model and the <3 years c-statistic score of the HEMORR2HAGES model was included because these are the c-statistic scores that match best with the original prediction window of both prediction models.

### Calibration

Eleven studies reported the predicted risk as event rate, which we recalculated to cumulative incidence at the prediction horizon as defined in the development study,^35^^,^^42^, ^43^, ^44^, ^45^, ^46^, ^47^, ^48^, ^49^, ^50^, ^51^ 1 study reported a cumulative incidence at the prediction timeframe of the development studies,^52^ and 4 studies reported a cumulative incidence at a different prediction timeframe, for which we approximated the cumulative incidence at the relevant timeframe from a Kaplan-Meier graph^31^^,^^41^^,^^53^^,^^54^ (Tables S3-S7). The reported cumulative incidence values differ between studies, which can be partly explained by the way of reporting. Some studies reported cumulative incidence values per score stratum, though most studies presented an aggregated observed risk for multiple score strata. Cumulative incidence rates and IS and bleeding outcomes per anticoagulant class were available for 10 studies (Table S8). In total, the IS and bleeding incidences were 1,779 (10.6%) and 795 (3.3%) in 16,755 patients for direct OAC/new OAC users and 7,162 (9.8%) and 3,521 (4.2%) in 73,455 patients for warfarin, aspirin, phenprocoumon, and vitamin K antagonist users, showing OAC versus warfarin hazard ratios ranging from 0.61-1.21 for IS and from 0.23-0.94 for bleeding.

## Meta-Analysis

### Discrimination

#### Discrimination in Patients With CKD

**Figure 2:**
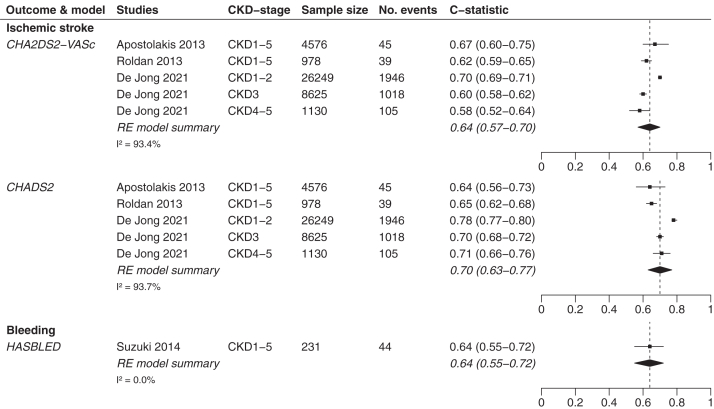
Forest plots of patients with CKD. CKD, chronic kidney disease; RE, random-effects. Forest plots of the HEMORR_2_HAGES model are not shown because the included studies did not contain any c-statistic scores on the HEMORR_2_HAGES model in patients with CKD.

For patients with CKD, the pooled discrimination results of IS prediction showed a pooled c-statistic score of 0.64 (95% CI, 0.57-0.70) for the CHA_2_DS_2_-VASc model and 0.70 (95% CI, 0.63-0.77) for the CHADS_2_ model (Fig 2). The pooled c-statistic scores are based on 5 studies, including 41,558 patients showing 3,153 events of IS. One study validated the HAS-BLED model, including 231 patients with CKD with 44 bleeding events, showing a c-statistic score of 0.64 (95% CI, 0.55-0.72). Pooled c-statistic scores could not be compared for bleeding risk models because of the absence of validations of the HEMORR_2_HAGES model in this population.

#### Discrimination in Patients Undergoing Dialysis

**Figure 3:**
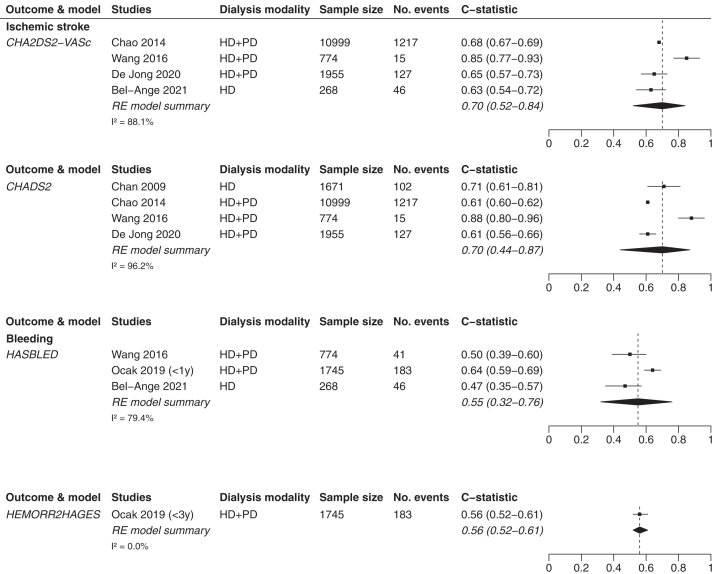
Forest plots of patients undergoing dialysis. HD, hemodialysis; PD, peritoneal dialysis; RE, random-effects.

For patients undergoing dialysis, the CHA_2_DS_2_-VASc and CHADS_2_ models performed similarly regarding IS prediction, with c-statistic scores of 0.70 (95% CI, 0.52-0.84) versus 0.70 (95% CI, 0.44-0.87), respectively. These pooled analyses are based on 4 studies, including 13,996 patients with 1,405 IS events for the CHA_2_DS_2_-VASc model and 15,399 patients with 1,461 IS events for the CHADS_2_ model. In patients undergoing dialysis, the HAS-BLED and HEMORR_2_HAGES models also performed similarly regarding bleeding prediction, with c-statistic scores of 0.55 (95% CI, 0.32-0.76) versus 0.56 (95% CI, 0.52-0.61), respectively (Fig 3). This is based on 3 studies on the HAS-BLED model, including 2,787 patients with 270 bleeding events, and 1 study on the HEMORR_2_HAGES model, including 1,745 patients with 183 bleeding events.

#### Discrimination in Patients With CKD and Patients Undergoing Dialysis Combined

**Figure 4:**
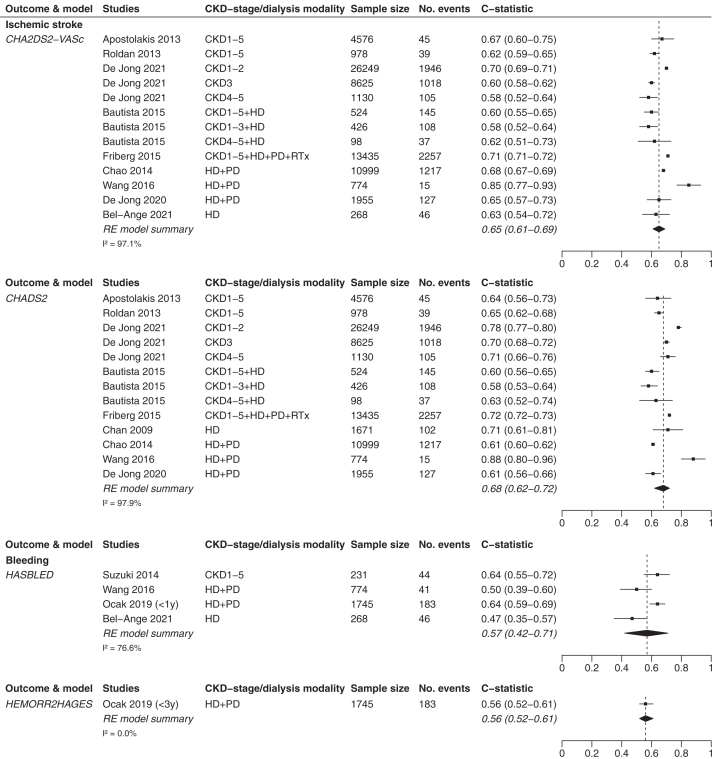
Forest plots of patients with CKD and patients undergoing dialysis combined. CKD, chronic kidney disease; HD, hemodialysis; PD, peritoneal dialysis; RE, random-effects; RTx, renal transplantation.

For patients with CKD and patients undergoing dialysis combined, the pooled discrimination results of IS prediction showed a pooled c-statistic score of 0.65 (95% CI, 0.61-0.69) for the CHA_2_DS_2_-VASc model and 0.68 (95% CI, 0.62-0.72) for the CHADS_2_ model. These pooled analyses are based on 9 studies, including 70,037 and 71,440 patients, showing 7,105 and 7,161 IS events for the CHA_2_DS_2_-VASc and CHADS_2_ models, respectively. In this combined group, the HAS-BLED and HEMORR_2_HAGES models performed similarly for bleeding prediction with a c-statistic score of 0.57 (95% CI, 0.42-0.71) versus 0.56 (95% CI, 0.52-0.61), respectively (Fig 4). This is based on 4 studies on the HAS-BLED model, including 3,018 patients showing 314 bleeding events, and 1 study on the HEMORR_2_HAGES model, including 1,745 patients showing 183 bleeding events.

### Calibration

**Figure 5:**
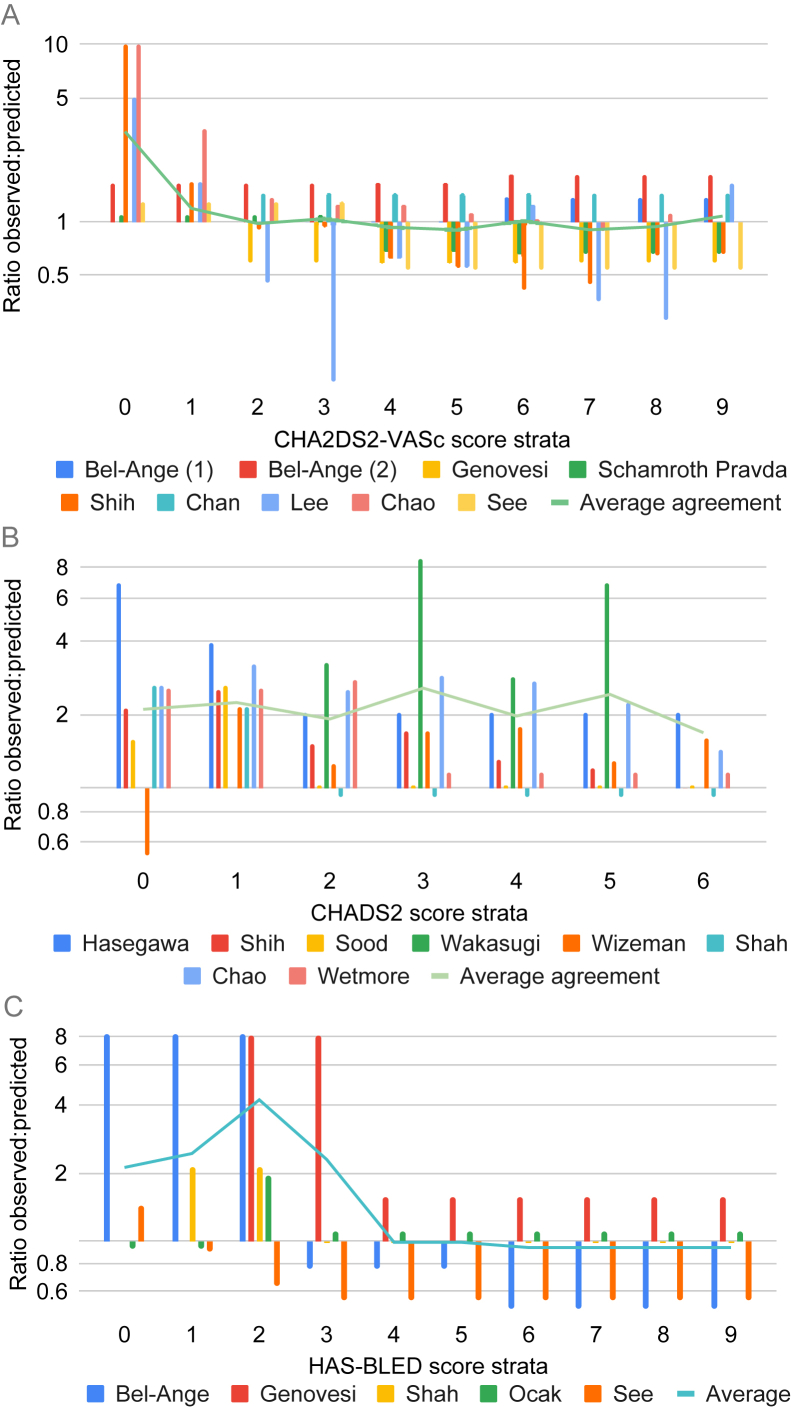
Agreement of the predictive and observed risks. (A) Upper panel: CHA_2_DS_2_-VASc model (stroke), (B) Middle panel: CHADS_2_ model (stroke), and (C) Lower panel: HAS-BLED model (bleeding) in patients undergoing dialysis (all types combined). There was insufficient data to aggregate the HEMORR_2_HAGES model in patients undergoing dialysis or any of the models in patients with CKD. A value of 1 on the y-axis indicates equal average observed and predicted rates. Values <1 indicate overprediction by the risk score compared with the observed risks, and vice versa for values >1. Ratios are calculated by (1) approximating observed event rates in validation studies, (2) predicting event rates in the development studies to cumulative incidence at the relevant prediction window, (3) calculating an unweighted mean observed cumulative incidence per score stratum for validation studies presenting aggregated observed risks for multiple score strata, and (4) comparing this to the unweighted mean predicted risks of these relevant score strata. The green line indicates the unweighted average of the ratio of observed versus predicted risks of all validation studies per risk stratum. Calibration plots of each validation study are presented in Figs S7-S9.

Figure 5 shows the agreement between the observed and predicted risks for both patients with CKD and patients undergoing dialysis in all types of AF. The CHA_2_DS_2_-VASc model (data available for 9 studies) showed underprediction (lower predicted than observed risks) for lower scores (0-1) and stable average agreement for scores of 2-9 (Fig 5A). The CHADS_2_ model (data available for 8 studies) showed stable underprediction for all risk strata (Fig 5B). The HAS-BLED model (data available for 5 studies) showed underprediction for scores of 0-3, but stable average agreement for scores of 4-9 (Fig 5C). Figures S7-S9 show an overview of the calibration plots.

### Sensitivity Analyses

Sensitivity analyses 1-6 showed a similar to nominally better performance of the CHADS_2_ model compared with the CHA_2_DS_2_-VASc model for IS prediction, in line with our main analysis. Only in patients undergoing dialysis with prevalent or unclear type AF (a subgroup analysis of sensitivity analysis 4), the CHA_2_DS_2_-VASc model performed nominally better than the CHADS_2_ model. In accordance with our main analysis, the 6 sensitivity analyses showed a similar performance of the HAS-BLED and HEMORR_2_HAGES models for bleeding prediction when enough data were available to draw forest plots. A more detailed overview of these results and their interpretation can be found in Figs S1-S6 and Supplement Section B: Interpretation of Sensitivity Analyses.

### ROB

**Figure 6:**
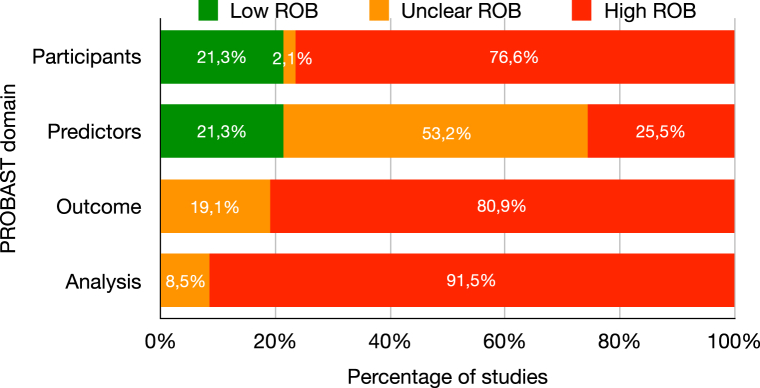
PROBAST tool summary. PROBAST, Prediction model Risk Of Bias ASsessment Tool.

The overall ROB score using the PROBAST tool was high (Fig 6 and Tables S9 and S10). There were high ROB scores in 77% of the Participants domains, 26% of the Predictors domains, 81% of the Outcome domains, and 92% of the Analysis domains. All Outcome and Analysis domains (eg, evaluating the definitions and determination of outcomes and the presence of relevant model performance measures) were at unclear or high ROB. The Predictors domain contained the highest number of studies with a low or unclear ROB score and the lowest number of studies with a high ROB score. The small-study effect was not evident from the funnel plots (Fig S10).

## Discussion

In this systematic review and meta-analysis, we demonstrated modest predictive abilities of the most commonly used prediction models for IS and bleeding in patients with AF undergoing dialysis or with CKD. In patients with AF and CKD, the CHADS_2_ model showed a slightly higher discriminative ability for predicting IS than the CHA_2_DS_2_-VASc model, whereas in dialysis patients, these models performed comparably. For bleeding prediction, the HAS-BLED and HEMORR_2_HAGES models performed similarly in patients with AF undergoing dialysis, whereas no conclusions can be drawn for patients with CKD because of a lack of studies. Calibration was poor in the low-risk groups, but the agreement between the observed and predicted risks was better in the clinically more relevant high-risk groups. All included validation studies were at high ROB scores, especially in the Outcome and Analysis domains.

### Clinical Implications of Findings

The benefit of anticoagulation in patients with AF undergoing dialysis or with CKD is a debated topic because these patients are at high risk for both IS and therapy-related bleeding, and guidelines provide little guidance in the context of CKD or dialysis.^1^, ^2^, ^3^, ^4^, ^5^, ^6^, ^7^, ^8^, ^9^, ^10^, ^11^, ^12^, ^13^, ^14^, ^15^, ^16^, ^17^, ^18^, ^19^, ^20^, ^21^, ^22^, ^23^, ^24^, ^25^, ^26^, ^27^, ^28^, ^29^, ^30^, ^31^, ^32^, ^33^, ^34^, ^35^, ^36^, ^37^^,^^55^ Most studies identified in our review explored the predictive performance of models predicting IS, reflecting the emphasis of AF guidelines on this topic.^7^ However, it is the high risk of bleeding that makes physicians hesitate to prescribe OACs to their patients with CKD or patients undergoing dialysis.^16^ Our study aggregated the available data on clinically relevant levels: IS and bleeding in early versus late CKD and dialysis—the last groups being usually excluded from trials.^55^^,^^56^ Overall, the discriminative abilities of the best-performing risk scores were modest but comparable to the results of these prediction models in patients with AF without CKD or without undergoing dialysis, which showed similar discrimination in large meta-analyses (pooled c-statistic scores of 0.644 [95% CI, 0.635-0.653] for the CHA_2_DS_2_-VASc model, 0.658 [95% CI, 0.644-0.672] for the CHADS_2_ model, 0.65 [95% CI, 0.61-0.69] for the HAS-BLED model, and 0.63 [95% CI, 0.61-0.66] for the HEMORR_2_HAGES model^14^^,^^15^). The poor calibration in low-risk groups has limited clinical relevance, as the proportion of patients with CKD classified as low risk is small according to the KDIGO guideline, because the high prevalence of comorbidities such as hypertension typically results in an elevated CHADS_2_ score.^57^ The better agreement between the observed and predicted risks for the CHA_2_DS_2_-VASc and the HAS-BLED models indicates that IS and bleeding risks can be weighed in these clinically more relevant higher-risk groups, which aligns with the KDIGO 2024 guideline recommendations to manage patients with both AF and CKD in the same manner as those without CKD.^57^

### Quality of the Main Findings

The ROB score assessment demonstrated that the included studies were all at high ROB, with especially the Outcome and Analysis domain being at high ROB. This finding is, however, prevalent in external validations of general AF^15^ and prediction research in general.^25^^,^^58^ Still, high ROB may lead to flawed or distorted conclusions regarding the predictive performance of prediction models,^58^ underlining the need for methodologically more rigorous validations. However, some nuance is needed in this context. The PROBAST, as a tool for assessing ROB score in prediction studies, has been criticized for being complex and subjective,^58^^,^^59^ which likely contributes to the low interrater agreement observed.^25^^,^^58^ Additionally, the PROBAST suffers from a ceiling effect, failing to differentiate between degrees of high ROB scores.^25^ Moreover, we encountered within-study and between-study heterogeneity (eg, in mixed patient cohorts and outcome definitions), showing I^2^ values of 76.6%-97.9% for our main analysis, which may explain the large CIs of some of the pooled estimates. However, because the direction of the observed effect was consistent across all analyses and aligned with findings in patients with AF without CKD, this heterogeneity can be seen as unlikely to be clinically meaningful.^60^ Finally, prediction windows were often not reported, although prediction models should preferably be validated using the same prediction window as the development study (ie, 1 year for the CHA_2_DS_2_-VASc and HAS-BLED model scores and 2 years and 8 months (ie, censoring after 1,000 days) for the HEMORR_2_HAGES and CHADS_2_ model scores^10^, ^11^, ^12^, ^13^), because the observed risk increases with time, influencing a model’s calibration. Despite their limitations, these studies were included in our analyses because they contain the best available evidence on IS and bleeding prediction in patients with AF undergoing dialysis or with CKD.

### Strengths and Limitations

Strengths of this study are the thorough search and the independent selection of articles by 2 reviewers, showing near-perfect agreement. Also, all 3 AF cohorts (incident, prevalent, and unclear type) and all clinically relevant levels of CKD are represented in the included studies. Lastly, this article includes a ROB score assessment by 2 independent reviewers using the PROBAST tool, thereby increasing the reliability of the reported ROB scores. This study has a number of limitations. First, the methodological quality of the included studies was modest to poor, as described above. Next, included studies used different validation strategies by combining scoring categories, impeding their comparability. Additionally, c-statistics were often aggregated for multiple CKD stages (eg, for stages 1-3, 1-5, or 4-5), making it impossible to pool c-statistic scores and create forest plots in the clinically more relevant late-stage CKD groups. The same holds true for the stratification of HD and PD. Because we only studied patients with CKD and patients undergoing HD and PD, generalizability to, for example, patients with different dialysis modalities or kidney transplant patients is limited, requiring more targeted validations of these populations. Moreover, the number of identified validation studies was relatively low, especially for the HEMORR_2_HAGES, reducing the reliability of these pooled results. Besides that, for the calibration plots, the observed risks of the validation studies should ideally be compared with the predicted risks of the development studies (ie, the risks as predicted using the risk score itself or the model behind it), but these were unavailable. We therefore used the observed risks of the development studies as a proxy for these predicted risks, as done before.^31^ Furthermore, many studies reported an aggregated risk per score stratum, necessitating comparison of observed and predicted unweighted arithmetic means, assuming both equal distribution of patients per risk score stratum and linearity. Next, most studies defined IS as ‘acute focal neurological symptoms’ lasting for a period of time. Because a substantial number of the included studies also validated bleeding models and confirmed their diagnoses by computed tomography scans or magnetic resonance imaging scans, we interpreted this outcome as IS. The outcome definitions of bleeding varied between studies, which is a problem not unique to our setting but broader, with only a small number of studies reporting this outcome in accordance with the criteria of the International Society on Thrombosis and Haemostasis.^61^ Finally, like most prediction studies, this study was based on mostly retrospective observational data, including studies where clinicians prescribed OACs before applying a prediction model, thereby possibly decreasing their discriminative value.

### Directives for Further Research

Although improving risk prediction in patients with CKD and patients undergoing dialysis is crucial, the first step should be to conduct validation studies with methodologically rigorous approaches. Initiatives, such as the PROGnosis RESearch Strategy (PROGRESS) for model development, the Transparent Reporting of a multivariable prediction model for Individual Prognosis Or Diagnosis (TRIPOD) guidelines for transparent reporting, and the PROBAST tool, may facilitate this. Reporting results stratified per CKD stage or dialysis modality allows for more precise evaluation of model performance across kidney function. There is limited evidence on the added value of CKD or dialysis-specific predictors in predicting IS and bleeding. Models including predictors such as estimated glomerular filtration rate or proteinuria did not show improved predictive performance.^40^ Validations of the R_2_CHADS_2_ model, an update of the CHADS_2_ including renal disease as a predictor, yield conflicting results.^36^^,^^62^ Further research on the added value of CKD- or dialysis-specific risk factors, such as the type of primary kidney disease, dialysis modality, or biomarkers specific for IS in CKD, is urgently needed both for IS and bleeding risk prediction.^63^ Furthermore, advanced and novel modeling methods such as machine learning may offer better performance by integrating a wider range of patient-specific data and addressing the complexities of the CKD and dialysis population.

## Conclusion

In patients with AF undergoing dialysis or with CKD, the CHA_2_DS_2_-VASc, CHADS_2_, HAS-BLED, and HEMORR_2_HAGES model scores showed modest discrimination, consistent with discrimination in patients with AF with normal kidney function. Calibration was poor in lower-risk groups but showed better agreement in higher-risk groups. Despite the identified ROB and heterogeneity, we believe these models are applicable and can be effectively used in clinical practice for patients with CKD and patients undergoing dialysis.

## Supplementary Materials

Supplementary File (PDF) Figures S1-S10; Items S1-S2; Tables S1-S10
