# Supplementary material for: Systematic Review and Meta-analysis of the Predictive Performance of Stroke and Bleeding Prediction Models in Atrial Fibrillation Patients With Kidney Disease
Source: Kidney Med. 2025 Dec 11;8(2):101200. doi: 10.1016/j.xkme.2025.101200 (PMC12861232; doi:10.1016/j.xkme.2025.101200)
Supplement: Supplementary File (PDF) — Figures S1-S10; Items S1-S2; Tables S1-S10 [file mmc1.pdf]

**SECTION A: INCLUDED STUDIES**

**Item S1. Detailed search method**

We searched for studies using two approaches: (1) a search in Web of Science (WOS) to identify all studies citing the $CHA_2DS_2$-VASc, $CHADS_2$, HAS-BLED and $HEMORR_2HAGES$ and (2) a search in PubMed, MEDLINE, Embase, Emcare, PMC, Cochrane Library and Academic Search Premier, using these risk scores as search term.

Detailed reasons for exclusion were (1) irrelevance, (2) wrong population - no AF, (3) wrong population – no CKD, (4) wrong outcome – not IS or bleeding, (5) review, (6) missing abstract/full text or (7) no predictive aim of the scores. Articles written in any other language than English, Dutch or German were excluded.

<u>Search string for Web of Science</u>

TS=(("CHA2DS2-VASc" OR "CHADS2" OR "HAS-BLED" OR "HEMORR2HAGES") AND (Renal Dialysis OR Dialysis OR Hemodialysis OR Haemodialysis OR Hemodiafiltration OR Haemodiafiltration OR Chronic Kidney Failure OR Chronic Renal Insufficiency OR Chronic Renal Failure OR Chronic Kidney Insufficiency OR CKD OR Chronic Kidney OR Chronic Renal OR End Stage Kidney Disease OR End Stage Kidney OR End Stage Renal OR EndStage Renal OR EndStage Kidney OR ESRD))

<u>Search string for PubMed, MEDLINE, Embase, Emcare, PMC, Cochrane Library and Academic Search Premier</u>

(("CHA2DS2-VASc" OR "CHADS2" OR "HAS-BLED" OR "HEMORR2HAGES") AND (Renal Dialysis OR Dialysis OR Hemodialysis OR Haemodialysis OR Hemodiafiltration OR Haemodiafiltration OR Chronic Kidney Failure OR Chronic Renal Insufficiency OR Chronic Renal Failure OR Chronic Kidney Insufficiency OR CKD OR Chronic Kidney OR Chronic Renal OR End Stage Kidney Disease OR End Stage Kidney OR End Stage Renal OR EndStage Renal OR EndStage Kidney OR ESRD)

## Item S2. Format predefined data extraction form

General data extraction form

| Study | Type of study | Country | Race/ ethnicity | Number of patients | Mean/ median age | % Men | Follow-up time | Loss to follow-up | Prediction horizon | CKD stage/ HD/PD | Type of AF | Incident/ prevalent / unclear type AF | Outcome | IS/ bleeding definition | Anti-coagulation at baseline | Anti-coagulation during follow-up | Validated models | Events | Discrimi-nation | C-statistic + 95% CI | Standard error | P-value | Event rate | Calibra-tion |
|---|---|---|---|---|---|---|---|---|---|---|---|---|---|---|---|---|---|---|---|---|---|---|---|---|
| Study 1 | | | | | | | | | | | | | | | | | | | | | | | | |
| Study 2 | | | | | | | | | | | | | | | | | | | | | | | | |
| …. | | | | | | | | | | | | | | | | | | | | | | | | |

Data extraction form for studies presenting c-statistics

| CKD stage/ HD/PD | Type of AF | Study | Validated model | Outcome | Sample size | Event size | C-statistic | Lower limit 95% CI | Upper limit 95% CI |
|---|---|---|---|---|---|---|---|---|---|
| | | | | | | | | | |
| | | | | | | | | | |
| | | | | | | | | | |

**Table S1: Overview of included studies**

**Table S1a: General characteristics of included studies**

| CKD or dialysis | Author | Country | Type of study | Included prediction models | Outcome: bleeding | Outcome: IS | Definition bleeding | Definition IS | Mean/ median follow-up |
|---|---|---|---|---|---|---|---|---|---|
| CKD | Apostolakis [37] | UK[1] | Mulitcentre, randomised, open-label non-inferiority study | $CHA_2DS_2$-VASc<br>HAS-BLED | x | x | Major bleed = bleeding that was fatal, intracranial, affecting another critical anatomical site, or overt bleeding | Stroke = focal neurological deficit of sudden onset with a corresponding defect on brain imaging | 325 d |
| | Barashi[64] | Israel | Retrospective cohort study | $CHA_2DS_2$- VASc | | x | NA | NA | 2.7 y |
| | Barcia[65] | Spain | Retrospective observational cohort study | $CHA_2DS_2$- VASc<br>HAS-BLED | x | x | Major bleeding: (1) fatal bleeding; (2) symptomatic bleeding in a critical area or organ, (3) bleeding causing a decrease in the hemoglobin level or more or leading to a transfusion | NA | 4.3y |
| | Calderon[66] | Spain | Retrospective cohort study | $CHA_2DS_2$- VASc<br>HAS-BLED | x | x | NA | NA | 3.2 y |
| | De Jong[5] | Sweden | Validation study | $CHADS_2$<br>$CHA_2DS_2$-VASc | | x | NA | Hospital admission for IS | 1.9 y |
| | Eikelboom [67] | 36 countries | Blinded randomised controlled trial | $CHADS_2$ | x | x | Major bleed = clinically overt bleeding or bleeding that was fatal | Stroke = acute onset of focal neurologic symptoms lasting > 24 h verified by CT or MRI. | 1.1 y |
| | Hart[68] | USA<br>Canada | Unblinded randomized controlled trial | $CHADS_2$ | x | x | Neurologic deficit > 24 h with imaging | Neurologic deficit > 24 h with imaging | 1.1 y |
| | Jun[69] | Australia<br>Canada | Multicentre retrospective cohort study | $CHA_2DS_2$- VASc<br>HAS-BLED | x | x | Major bleeding = intracranial, upper or lower gastrointestinal, or other bleeding | Stroke = ischemic stroke, transient ischemic attack or systemic embolism | 1 y |

| CKD or dialysis | Author | Country | Type of study | Included prediction models | Outcome: bleeding | Outcome: IS | Definition bleeding | Definition IS | Mean/ median follow-up |
|---|---|---|---|---|---|---|---|---|---|
| | Kee[70] | Korea | Retrospective cohort study | $CHA_2DS_2$- VASc HAS-BLED[2] | x | x | NA | NA | 23.8 mo |
| | Li[71] | China | Prospective multicentre registry study | $CHA_2DS_2$- VASc HAS-BLED | x | x | Major bleeding = bleeding that was fatal, intracranial, affecting another critical anatomical site, or causing a fall in hemoglobin level, or leading to transfusion | Stroke = ischemic stroke, transient ischemic attack or systemic embolism | [3] |
| | McAlister[72] | Canada | Retrospective cohort study | $CHADS_2$ $CHA_2DS_2$-VASc | x | x | NA | NA | 31 mo |
| | Nakagawa[73] | Japan | Follow-up study | $CHADS_2$ | x | x | Neurologic deficit > 24 h with imaging | Neurologic deficit > 24 h with imaging | 5.6 y |
| | Ocak[3] | The Netherlands | Prospective singlecentre cohort study | $CHA_2DS_2$- VASc | x | x | Bleeding = fatal or non-fatal hemorrhagic event, confirmed by evaluation of hospital discharge records and results of relevant laboratory and radiology examinations. | IS = clinical features with increased impairment of ≥ 1 point on the modified Rankin Scale, without signs of hemorrhage on brain imaging (MRI or CT scan). | 6.4 y |
| | Rivera-Caravaca[74] | Spain | Observational study | $CHADS_2$ $CHA_2DS_2$- VASc HAS-BLED | x | x | NA | Stroke = ischemic stroke, transient ischemic attack or systemic embolism | 2.2 y |
| | Roldan[40] | Spain UK[1] | Prospective cohort study | $CHADS_2$ $CHA_2DS_2$-VASc | | x | NA | NA | 875 d |
| | Suzuki[38] | Japan | Prospective study | HAS-BLED | x | | Clinically overt, associated with a fall in hemoglobin level, involving a critical site, or fatal. | NA | 7.1 y |
| Dialysis | Akbar[75] | Indonesia | Singlecentre retrospective observational study | $CHA_2DS_2$- VASc HAS-BLED | | x | NA | Stroke = the presence of a focal neurologic deficit requiring hospitalization, and it was confirmed by computed | 11 mo |

| CKD or dialysis | Author | Country | Type of study | Included prediction models | Outcome: bleeding | Outcome: IS | Definition bleeding | Definition IS | Mean/ median follow-up |
|---|---|---|---|---|---|---|---|---|---|
| | | | | | | | | tomography or magnetic resonance imaging | |
| | Bel-Ange[41] | Israel[1] | Prospective cohort study | $CHA_2DS_2$-VASc HAS-BLED | x | x | Major bleed = any kind requiring hospitalisation or fatal bleeding | IS = radiological documentation by brain imaging studies | 21 mo |
| | Chan[53] | USA | Retrospective cohort study | $CHADS_2$ | | x | NA | NA | 1.6 y |
| | Chan[34] | China | Single centre observational study | $CHA_2DS_2$-VASc HAS-BLED | x | x | Bleeding = intracranial, new onset neurological symptoms with radiological confirmation | IS = Neurologic deficit > 24 h with imaging. | 18 m |
| | Chao[35] | Taiwan | Retrospective insurance database | $CHADS_2$ $CHA_2DS_2$-VASc | | x | NA | NA | 13 mo |
| | De Jong[23] | The Netherlands | Prospective cohort study | $CHADS_2$ $CHA_2DS_2$-VASc | | x | NA | IS requiring hospitalization or fatal IS | 2.5 y |
| | Elfar[76] | Egypt UK | Systematic review and meta-analysis | $CHA_2DS_2$- VASc | x | x | Major bleeding = bleeding in a critical area or organ; bleeding causing a drop in hemoglobin level; and/or requiring transfusion | IS = acute occlusion of an arterial vessel, excluding the heart, and brain | 106-540 d |
| | Genovesi[77] | Italy | Prospective cohort study | $CHA_2DS_2$-VASc HAS-BLED | x | x | NA | Stroke = ischemic stroke, transient ischemic attack or systemic embolism | 4 y |
| | Hasegawa[43] | Japan | Observational prospective cohort study | $CHADS_2$ | | x | NA | Hospitalised for stroke | [3] |
| | Kim[78] | Korea | Singlecentre retrospective cohort study | $CHA_2DS_2$- VASc HAS-BLED | x | x | Bleeding = gastrointestinal bleeding with admission or cerebrovascular bleeding or other life-threatening bleeding | NA | 879 d |
| | Lee[48] | Taiwan | Population-based cohort study | $CHA_2DS_2$-VASc | | x | NA | NA | 3.2 y |

| CKD or dialysis | Author | Country | Type of study | Included prediction models | Outcome: bleeding | Outcome: IS | Definition bleeding | Definition IS | Mean/ median follow-up |
|---|---|---|---|---|---|---|---|---|---|
| | Ocak[31] | The Netherlands | Prospective cohort study | HAS-BLED<br>$HEMORR_2HAGES$ | x | | Bleeding requiring hospitalization or fatal within 3y of FU | NA | 2.2 y |
| | Pokorney[79] | USA | Prospective cohort study | $CHA_2DS_2$- VASc | x | x | Major bleeding event = acute, clinically overt bleeding event with a decrease in hemoglobin, a transfusion, or bleeding within a critical site. Any hemorrhagic stroke, defined as primary hemorrhagic or infarction with hemorrhagic conversion | Stroke = abrupt onset of focal neurological symptoms lasting ≥24 hours. Imaging studies were used to support the stroke diagnosis | [3] |
| | Reinecke[80] | Germany | Randomised controlled trial | $CHA_2DS_2$- VASc<br>HAS-BLED | x | x | NA | NA | 462 d |
| | Schamroth Pravda[52] | Israel | Retrospective, single center | $CHA_2DS_2$-VASc | | x | NA | NA | 955 d |
| | See[50] | Taiwan | Population-based cohort study | $CHA_2DS_2$-VASc | x | x | NA | NA | NA |
| | Shah[49] | Canada | Population based retrospective cohort study | $CHADS_2$<br>HAS-BLED[2] | x | x | Bleeding = intracerebral, gastrointestinal, intraocular, hematuria and unspecified location of bleeding | Stroke = ischemic cerebrovascular disease, including TIA and retinal infarct. | NA |
| | Shih[44] | Taiwan | Retrospective, insurance database | $CHA_2DS_2$-VASc | | x | NA | NA | 3.2 y |
| | Sood[45] | 12 countries | Prospective cohort study | $CHADS_2$ | x | x | NA | Stroke requiring hospitalization | [3] |
| | Wakasugi[46] | Japan | Prospective, multicenter cohort study | $CHADS_2$ | x | x | Neurologic deficit > 24 h with imaging | Neurologic deficit > 24 h with imaging | 110 py |
| | Wang[33] | New-Zealand | Retrospective, single center | $CHA_2DS_2$-VASc | x | x | Neurologic deficit > 24 h with imaging | Neurologic deficit > 24 h with imaging | 3.4 y |
| | Wetmore[54] | USA | Retrospective cohort analysis | $CHADS_2$ | | x | NA | NA | 18.7 mo |

| CKD or dialysis | Author | Country | Type of study | Included prediction models | Outcome: bleeding | Outcome: IS | Definition bleeding | Definition IS | Mean/ median follow-up |
|---|---|---|---|---|---|---|---|---|---|
| | Wetmore[81] | USA | Retrospective cohort study | $CHA_2DS_2$- VASc HAS-BLED | x | x | NA | Stroke = ischemic stroke, transient ischemic attack or systemic embolism | NA |
| | Wizemann[47] | 12 countries | DOPPS I + II | $CHADS_2$ | | x | NA | Stroke requiring hospitalization | NA |
| | Xu[82] | Australia | Retrospective cohort study | $CHADS_2$ $CHA_2DS_2$-VASc | | x | NA | Radiologic diagnosis on MRI or clinical diagnosis | 526 py |
| CKD and dialysis combined | Bautista[36] | USA | Retrospective cohort study | $CHADS_2$ $CHA_2DS_2$-VASc | | x | NA | NA | NA |
| | Friberg[39] | Sweden | Retrospective health registers | $CHADS_2$ $CHA_2DS_2$-VASc | x | x | NA | NA | 2.1 y |
| | Park[83] | South Korea | Prospective observational registry study | $CHA_2DS_2$- VASc HAS-BLED | x | x | NA | Stroke/systemic embolism included CVA, TIA, and acute loss of blood flow to a peripheral artery confirmed by MRI or CT | 24 mo |
| | Sy[51] | USA | Retrospective cohort study | $CHA_2DS_2$- VASc HAS-BLED | x | x | NA | NA | NA |
| | Welander[85] | Sweden | Cohort study | $CHA_2DS_2$- VASc | x | x | NA | NA | NA |
| | Welander[84] | Sweden | Retrospective cohort study | $CHA_2DS_2$- VASc | x | x | NA | NA | 2.2 y |

Abbreviations: CKD = chronic kidney disease, NA = not available or not applicable, IS = ischemic stroke, MRI = magnetic resonance imaging, CT = computer tomography, CVA = cerebrovascular accident, TIA = transient ischemic attack, d = days, w = weeks, mo = months, y= years, py = person years

[1] Based on authors place of work
[2] Modified HAS-BLED with a maximum score of 7 instead of 9
[3] Information available per group in the population

**Table S1b: Demographic data of included studies**

| | | Kidney function | | | | | | Atrial fibrillation | Anticoagulation | | Demographic information | | | | |
|---|---|---|---|---|---|---|---|---|---|---|---|---|---|---|---|
| | | CKD stage | | | | | dialysis | | | | | | | | |
| | | 1 | 2 | 3 | 4 | 5 | (type of dialysis) | Type of AF | Baseline | Follow-up | Mean/ median age (years) | % male | Ethnicity | N | Events (%) |
| CKD | Apostolakis[37] | x | x | x | x | | | Prevalent | Idraparinux, VKA | Idraparinux, VKA | 70 | 66.5 | NA | 4576 | 45 (1.0) IS<br>103 (2.3) bleed |
| | Barashi[64] | x | x | x | x[1] | x[1] | | Prevalent | DOAC, aspirin | DOAC | 77.2 | 46.8 | NA | 19 713 | 2295 (11.6) IS |
| | Barcia[65] | x | x | x | x[1] | x[1] | | Incident | DOAC, VKA, LMWH | DOAC, VKA, LMWH | [2] | 49.3 | NA | 15 457 | 850 (5.5) IS<br>961 (6.2) bleed |
| | Calderon[66] | x | x | x | x | | | Incident & prevalent | VKA, NOAC, aspirin | VKA, NOAC, aspirin | 73.3 | 47 | NA | 65 734 | 8592 (13.1) IS<br>2440 (3.7) bleed |
| | De Jong[5] | x | x | x | x | x | | Incident | VKA, DOAC, antiplatelets | None | 74.8 | 52.5 | Caucasian | 36 004 | 3069 (8.5) IS |
| | Eikelboom[67] | x | x | x[3] | | | | Prevalent | Unclear | Apixaban, aspirin | 70 | 59 | Caucasian<br>Others | 5525 | NA |
| | Hart[68] | x | x | x | | | | Unclear type | Unclear | Warfarin, aspirin, aspirin+ low dose warfarin | 70 | [2] | Caucasian<br>African-American<br>Hispanic | 1936 | NA |

| | | Kidney function | | | | | | Atrial fibrillation | Anticoagulation | | Demographic information | | | | |
|---|---|---|---|---|---|---|---|---|---|---|---|---|---|---|---|
| | | CKD stage | | | | | dialysis | | | | | | | | |
| | | 1 | 2 | 3 | 4 | 5 | (type of dialysis) | Type of AF | Baseline | Follow-up | Mean/ median age (years) | % male | Ethnicity | N | Events (%) |
| | | | | | | | | | | | | | Other | | |
| | Jun[69] | x | x | x | x[1] | x[1] | | Unclear type | Antiplatelets or no antiplatelets | DOAC, warfarin | 75.1 | 54.3 | NA | 174<br>157 | 875 (0.5) IS<br>1572 (0.9) bleed |
| | Kee[70] | x | x | x | x | x | | Incident | None | DOAC, warfarin | 71.1 | 56.2 | NA | 1885 | 293 (15.5) IS<br>483 (25.6) bleed |
| | Li[71] | x | x | x | x[1] | x[1] | | Prevalent | DOAC, warfarin, antiplatelet | DOAC, warfarin, antiplatelet | 63.9 | 62.1 | NA | 19 079 | 985 (5.6) IS<br>414 (2.2) bleed |
| | McAlister[72] | [4] | [4] | [4] | [4] | [4] | | Incident | None | None | 66 | 53 | NA | 18295 | 7340 (40.1) IS<br>4625 (25.3) bleed |
| | Nakagawa[73] | x | x | x | x | x | | Prevalent | Antiplatelets, warfarin | Antiplatelets, warfarin | 66 | 75 | NA | 387 | 25 (6.5) IS<br>6 (1.6) bleed |
| | Ocak[3] | [2] | [2] | [2] | [2] | [2] | | Prevalent | Antiplatelets, other antcoagulant | Antiplatelets, other anticoagulant | 71[5] | 72[5] | NA | 325[5] | 46 (14.2) IS<br>37 (11.4) bleed |
| | Rivera-Caravaca[74] | x | x | x | x | x | | Prevalent | Rivaroxaban, antiplatelets | Rivaroxaban, antiplatelets | 74.2 | 55.5 | NA | 1433 | 21 (1.5) IS<br>33 (2.3) bleed |
| | Roldan[40] | x | x | x | x | x | | Prevalent | VKA | VKA | 76 | 51 | NA | 978[6] | 39 (4.0) IS |
| | Suzuki[38] | x | x | x | x | x | | Prevalent | Antiplatelets | Warfarin | [2] | [2] | NA | 231 | 44 (19.0) bleed |
| Dialysis | Akbar[75] | | | | | | x (HD) | Prevalent | Warfarin | Warfarin | 52.2 | 52.3 | NA | 88 | 9 (10.2) IS |
| | Bel-Ange[41] | | | | | | x (HD) | Prevalent | Warfarin, aspirin, clopidogrel, enoxaparin, DAT | Warfarin, aspirin, clopidogrel, enoxaparin, DAT | 73.4 | 39 | NA | 268 | 46 (17.2) IS<br>24 (9.0) bleed |
| | Chan[53] | | | | | | x (HD) | Prevalent | Warfarin, clopidogrel, aspirin | Warfarin, clopidogrel, aspirin | [2] | [2] | White<br>Black<br>Other | 1671 | 102 (6.1) IS |

| | | **Kidney function** | | | | | | **Atrial fibrillation** | **Anticoagulation** | | **Demographic information** | | | | |
|---|---|---|---|---|---|---|---|---|---|---|---|---|---|---|---|
| | | **CKD stage** | | | | | **dialysis** | | | | | | | | |
| | | 1 | 2 | 3 | 4 | 5 | (type of dialysis) | Type of AF | Baseline | Follow-up | Mean/ median age (years) | % male | Ethnicity | N | Events (%) |
| | Chan[34] | | | | | | x (PD) | Incident | Warfarin, aspirin | Warfarin, aspirin | 76.8 | 48 | NA | 271 | 19 (7.0) IS<br>2 (0.7) bleed |
| | Chao[35] | | | | | | x (HD & PD) | Prevalent | None | None | 71.0 | 46.2 | NA | 10 999 | 1217 (11.1) IS |
| | De Jong[23] | | | | | | x (HD & PD) | Unclear type | VKA, antiplatelets | VKA, antiplatelets | 59.9 | 62.2 | NA | 1955 | 127 (6.5) IS |
| | Elfar[76] | | | | | | x (HD) | Prevalent | VKA, DOAC | VKA, DOAC | [2] | [2] | NA | 34 516 | 250 (0.7) IS<br>1167 (3.4) bleed |
| | Genovesi[77] | | | | | | x (HD) | Prevalent | Warfarin, antiplatelets | Warfarin, antiplatelets | 74 | 60 | NA | 290 | 28 (9.7) IS<br>95 (32.8) bleed |
| | Hasegawa[43] | | | | | | x (HD) | Incident & prevalent | Warfarin, aspirin, antiaggregant | Warfarin, aspirin, antiaggregant | [2] | [2] | NA | 399[5] | NA |
| | Kim[78] | | | | | | x (HD & PD) | Unclear type | Apixaban, warfarin, aspirin, clopidogrel | Warfarin, apixaban | [2] | [2] | NA | 89 | 8 (9.0) IS<br>16 (18.0) bleed |
| | Lee[48] | | | | | | x (PD) | Incident | Clopidogrel+ aspirin | Warfarin | 63.1 | 49.5 | NA | 505 | 43 (8.5) IS |
| | Ocak[31] | | | | | | x (HD & PD) | Unclear type | VKA | VKA | 62 | 61 | NA | 1745 | 183 (10.5) bleed |
| | Pokorney[79] | | | | | | x (HD) | Prevalent | Warfarin, non-VKA | Apixaban, warfarin | 68 | 44 | Black<br>Non-black | 154 | 3 (1.9) IS<br>37 (24.0) bleed |
| | Reinecke[80] | | | | | | x (HD) | Prevalent | Aspirin, VKA, NOAC | Apixaban, phenprocoumon | 74.7 | 68 | NA | 97 | 1 (1.0) IS<br>11 (11.3) bleed |

| | | **Kidney function** | | | | | | **Atrial fibrillation** | **Anticoagulation** | | **Demographic information** | | | | |
|---|---|---|---|---|---|---|---|---|---|---|---|---|---|---|---|
| | | **CKD stage** | | | | | **dialysis** | | | | | | | | |
| | | 1 | 2 | 3 | 4 | 5 | (type of dialysis) | Type of AF | Baseline | Follow-up | Mean/ median age (years) | % male | Ethnicity | N | Events (%) |
| | Schamroth Pravda[52] | | | | | | x (HD) | Unclear type | Aspirin | Aspirin | 66 | 63.9 | NA | 457 | 17 (3.7) IS |
| | See[50] | | | | | | x (NA) | Incident | Warfarin, NOAC | Warfarin, NOAC | [2] | [2] | NA | 12500 | [2] |
| | Shah[49] | | | | | | x (HD & PD) | Incident | Aspirin, clopidogrel | Warfarin | [2] | [2] | NA | 1626[5] | 107 (6.6) IS<br>275 (16.9) bleed |
| | Shih[44] | | | | | | x (HD) | Incident | Unfractionated heparin | Warfarin | 68.8 | 46.8 | NA | 6772 | 600 (8.9) IS |
| | Sood[45] | | | | | | x (HD) | Prevalent | Warfarin, antiplatelets, acetylsalicylic acid | Warfarin, antiplatelets, acetylsalicylic acid | [2] | [2] | Blacks<br>Others | 37 898 | [2] |
| | Wakasugi[46] | | | | | | x (HD) | Prevalent | Warfarin, antiplatelets | Warfarin, antiplatelets | 68.1 | 65 | NA | 60 | 13 (21.7) IS<br>7 (11.7) bleed |
| | Wang[33] | | | | | | x (HD & PD) | Incident | Warfarin | Warfarin, clopidogrel, aspirin | 61.2 | 61.7 | Caucasian<br>Maori<br>Pacific Island<br>Asian | 141 | 15 (10.6) IS<br>41 (29.1) bleed |
| | Wetmore[54] | | | | | | x (NA) | Prevalent | Unclear | Unclear | 60.6 | 43.4 | African-American<br>Caucasian<br>Hispanic<br>Other | 56 734 | NA |
| | Wetmore[81] | | | | | | x (HD & PD) | Prevalent | Warfarin, apixaban, DOAC | Warfarin, apixaban, DOAC | 66.2 | 61.7 | Black<br>White<br>Other | 17 156 | 530 (3.1) IS<br>1470 (8.6) bleed |
| | Wizemann[47] | | | | | | x (HD) | Incident & prevalent | Warfarin, aspirin | Warfarin, aspirin | NA | NA | Blacks<br>Others | 17 513 | [2] |

| | | **Kidney function** | | | | | | **Atrial fibrillation** | **Anticoagulation** | | **Demographic information** | | | | |
|---|---|---|---|---|---|---|---|---|---|---|---|---|---|---|---|
| | | **CKD stage** | | | | | **dialysis** | | | | | | | | |
| | | 1 | 2 | 3 | 4 | 5 | (type of dialysis) | Type of AF | Baseline | Follow-up | Mean/ median age (years) | % male | Ethnicity | N | Events (%) |
| | Xu[82] | | | | | | x (HD) | Prevalent | Warfarin or no warfarin | Warfarin | [2] | 42.9 | Indigenous Australians<br>Non-indigenous Australians | 182 | 4 (2.2) IS |
| CKD and dialysis combined | Bautista[36] | x | x | x | x | x | x (HD) | Unclear type | Warfarin, heparin, aspirin | Warfarin, heparin, aspirin | [2] | [2] | Hispanic<br>African-America<br>Other (incl caucasian) | 524 | 145 (27.7) IS |
| | Friberg[39] | [4] | [4] | [4] | [4] | [4] | x (HD & PD) | Incident | Warfarin, fenprocoumon, aspirin | Warfarin, fenprocoumon, aspirin | 78.4 | 54.9 | NA | 13 435 | 2257 (16.8) IS<br>9001 (67.0) bleed |
| | Park[83] | | | | x | x | x (NA) | Prevalent | None | Warfarin, DOAC, no anticoagulation | [2] | [2] | NA | 260 | NA |
| | Sy[51] | | | | | x | x (HD) | Prevalent | Warfarin or no warfarin | Warfarin or no warfarin | 77 | 96 | White<br>African-American<br>Hispanic<br>Other | 28 620 | NA |
| | Welander[85] | | | x | x | x | x (HD & PD) | Prevalent | Warfarin, DOAC | Warfarin, DOAC | 77.3 | 68.4 | NA | 12 106 | 681 (5.6) IS<br>2297 (19.0) bleed |
| | Welander[84] | | | x | x | x | x (HD & PD) | Prevalent | Warfarin, DOAC | Warfarin, DOAC | 76.7 | 68.1 | NA | 2453 | 91 (3.7) IS<br>352 (14.3) bleed |

Abreviations: CKD = chronic kidney disease, NA = not applicable or not available, HD = hemodialysis, PD = peritoneal dialysis, VKA = vitamin K antagonist, DAT = dual antiplatelet therapy, NOAC = novel oral anticoaculation, DOAC = direct oral anticoagulation, LMWH = low molecular weight heparin, IS = ischemic stroke

[1] eGFR < 30, CKD stage 4 and 5 not named as separate groups
[2] Information available per group in the population
[3] eGFR < 25 excluded
[4] Information on stage of chronic kidney disease (CKD) not available
[5] AF + CKD/dialysis subgroup of the total studypopulation
[6] Of which 177 with a normal estimated glomerular filtration rate (eGFR)
[7] 6603 patients without atrial fibrillation (AF) and 399 patients with AF

**Table S2: Number of included studies, patients and events**

| Kidney function | Prediction model | Number of studies | Number of patients | Number of ischemic stroke events | Number of bleeding events |
|---|---|---|---|---|---|
| CKD | $CHA_2DS$-VASc | 12 | 357 636 | 24 450 | 10 668 |
| | $CHADS_2$ | 7 | 64 558 | 10 494 | 4664 |
| | HAS-BLED | 8 | 282 552 | 11 661 | 6050 |
| | $HEMORR_2HAGES$ | 0 | 0 | 0 | 0 |
| | Total CKD | 16 | 365 715 | 22 180 | 10 718 |
| Dialysis | $CHA_2DS$-VASc | 17 | 86 440 | 2917 | 2863 |
| | $CHADS_2$ | 10 | 129 037 | 1570 | 282 |
| | HAS-BLED | 9 | 21 630 | 748 | 2076 |
| | $HEMORR_2HAGES$ | 1 | 1745 | 0 | 183 |
| | Total dialysis | 25 | 204 086 | 3139 | 3328 |
| CKD+dialysis | $CHA_2DS$-VASc | 6 | 57 398 | 3174 | 11 649 |
| | $CHADS_2$ | 2 | 13 959 | 2402 | 9001 |
| | HAS-BLED | 2 | 28 880 | Not available | Not available |
| | $HEMORR_2HAGES$ | 0 | 0 | 0 | 0 |
| | Total CKD+dialysis | 6 | 57 398 | 3174 | 11 649 |
| TOTAL | | 47 | 627 199 | 28 493 | 25 695 |

## SECTION B: SENSITIVITY ANALYSES

**Figure S1: Forest plots sensitivity analysis 1**

Figure for CKD and dialysis combined. Separate figures for CKD only and dialysis only are not presented because these are identical to the CKD only and dialysis only figures of the main analysis (see article Figure 2 and Figure 3).

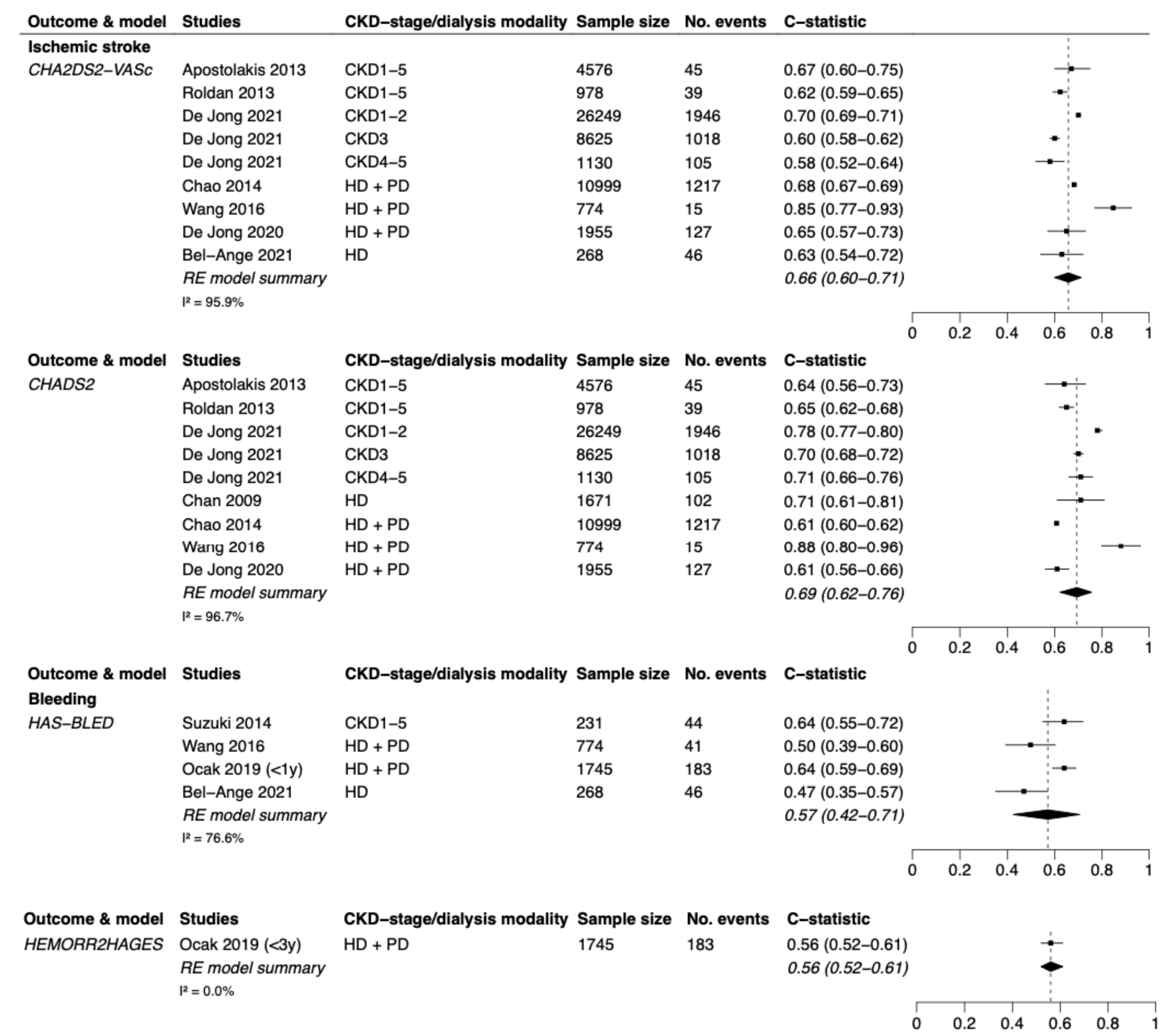

**Figure S2: Forest plots sensitivity analysis 2**

Figure for CKD and dialysis combined. Separate figures for CKD or dialysis only are unavailable due to the absence of studies on CKD only and dialysis only matching the criteria for this sensitivity analysis.

| Outcome & model | Studies | CKD−stage | Sample size | No. events | C−statistic |
|---|---|---|---|---|---|
| **Ischemic stroke** | | | | | |
| *CHA2DS2−VASc* | Bautista 2015 | CKD1−5+HD | 524 | 145 | 0.60 (0.55−0.65) |
| | Bautista 2015 | CKD1−3+HD | 426 | 108 | 0.58 (0.52−0.64) |
| | Bautista 2015 | CKD4−5+HD | 98 | 37 | 0.62 (0.51−0.73) |
| | Friberg 2015 | CKD1−5+HD+PD+RTx | 13435 | 2257 | 0.71 (0.71−0.72) |
| | *RE model summary* | | | | *0.63 (0.53−0.73)* |
| | I² = 88.6% | | | | |
| *CHADS2* | Bautista 2015 | CKD1−5+HD | 524 | 145 | 0.60 (0.56−0.65) |
| | Bautista 2015 | CKD1−3+HD | 426 | 108 | 0.58 (0.53−0.64) |
| | Bautista 2015 | CKD4−5+HD | 98 | 37 | 0.63 (0.52−0.74) |
| | Friberg 2015 | CKD1−5+HD+PD+RTx | 13435 | 2257 | 0.72 (0.72−0.73) |
| | *RE model summary* | | | | *0.64 (0.53−0.74)* |
| | I² = 91.2% | | | | |

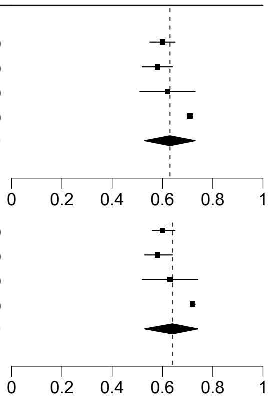

**Figure S3: Forest plots sensitivity analysis 3**

a) Forest plots for CKD patients only, b) forest plots for dialysis patients only and c) forest plots for CKD and dialysis patients combined.

| Outcome & model | Studies | CKD−stage | Sample size | No. events | C−statistic |
|---|---|---|---|---|---|
| **Ischemic stroke** | | | | | |
| *CHA2DS2−VASc* | De Jong 2021 | CKD1−2 | 26249 | 1946 | 0.70 (0.69−0.71) |
| | De Jong 2021 | CKD3 | 8625 | 1018 | 0.60 (0.58−0.62) |
| | De Jong 2021 | CKD4−5 | 1130 | 105 | 0.58 (0.52−0.64) |
| | *RE model summary* | | | | *0.63 (0.46−0.78)* |
| | I² = 97.3% | | | | |
| *CHADS2* | De Jong 2021 | CKD1−2 | 26249 | 1946 | 0.78 (0.77−0.80) |
| | De Jong 2021 | CKD3 | 8625 | 1018 | 0.70 (0.68−0.72) |
| | De Jong 2021 | CKD4−5 | 1130 | 105 | 0.71 (0.66−0.76) |
| | *RE model summary* | | | | *0.73 (0.61−0.83)* |
| | I² = 93.5% | | | | |

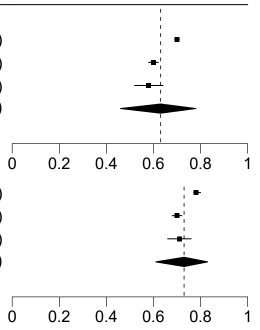


a)

| Outcome & model | Studies | Dialysis modality | Sample size | No. events | C−statistic |
|---|---|---|---|---|---|
| **Ischemic stroke** | | | | | |
| *CHA2DS2−VASc* | Wang 2016 | HD+PD | 774 | 15 | 0.85 (0.77−0.93) |
| | *RE model summary* | | | | *0.85 (0.77−0.93)* |
| | I² = 0.0% | | | | |
| *CHADS2* | Wang 2016 | HD+PD | 774 | 15 | 0.88 (0.80−0.96) |
| | *RE model summary* | | | | *0.88 (0.80−0.96)* |
| | I² = 0.0% | | | | |
| **Bleeding** | | | | | |
| *HAS−BLED* | Wang 2016 | HD+PD | 774 | 41 | 0.50 (0.39−0.60) |
| | *RE model summary* | | | | *0.50 (0.39−0.60)* |
| | I² = 0.0% | | | | |

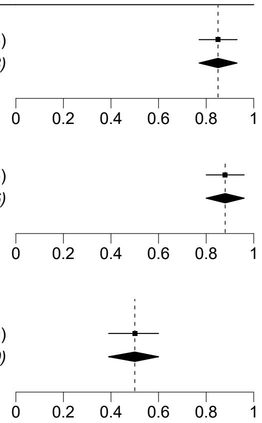


b)

| Outcome & model | Studies | CKD−stage/dialysis modality | Sample size | No. events | C−statistic |
|---|---|---|---|---|---|
| **Ischemic stroke** | | | | | |
| *CHA2DS2−VASc* | De Jong 2021 | CKD1−2 | 26249 | 1946 | 0.70 (0.69−0.71) |
| | De Jong 2021 | CKD3 | 8625 | 1018 | 0.60 (0.58−0.62) |
| | De Jong 2021 | CKD4−5 | 1130 | 105 | 0.58 (0.52−0.64) |
| | Friberg 2015 | CKD1−5+HD+PD+RTx | 13435 | 2257 | 0.71 (0.71−0.72) |
| | Wang 2016 | HD+PD | 774 | 15 | 0.85 (0.77−0.93) |
| | *RE model summary* | | | | *0.69 (0.54−0.80)* |
| | I² = 99.5% | | | | |
| *CHADS2* | De Jong 2021 | CKD1−2 | 26249 | 1946 | 0.78 (0.77−0.80) |
| | De Jong 2021 | CKD3 | 8625 | 1018 | 0.70 (0.68−0.72) |
| | De Jong 2021 | CKD4−5 | 1130 | 105 | 0.71 (0.66−0.76) |
| | Friberg 2015 | CKD1−5+HD+PD+RTx | 13435 | 2257 | 0.72 (0.72−0.73) |
| | Wang 2016 | HD+PD | 774 | 15 | 0.88 (0.80−0.96) |
| | *RE model summary* | | | | *0.74 (0.66−0.81)* |
| | I² = 96.0% | | | | |
| **Bleeding** | | | | | |
| *HAS−BLED* | Wang 2016 | HD+PD | 774 | 41 | 0.50 (0.39−0.60) |
| | *RE model summary* | | | | *0.50 (0.39−0.60)* |
| | I² = 0.0% | | | | |

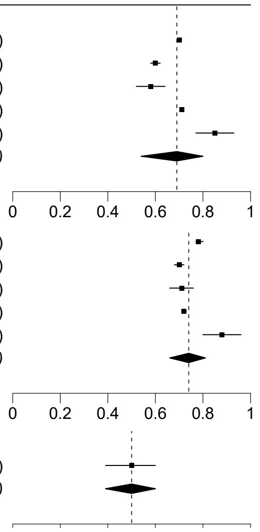


c)

**Figure S4: Forest plots sensitivity analysis 4**

a) Forest plots for CKD patients only, b) forest plots for dialysis patients only and c) forest plots for CKD and dialysis patients combined.

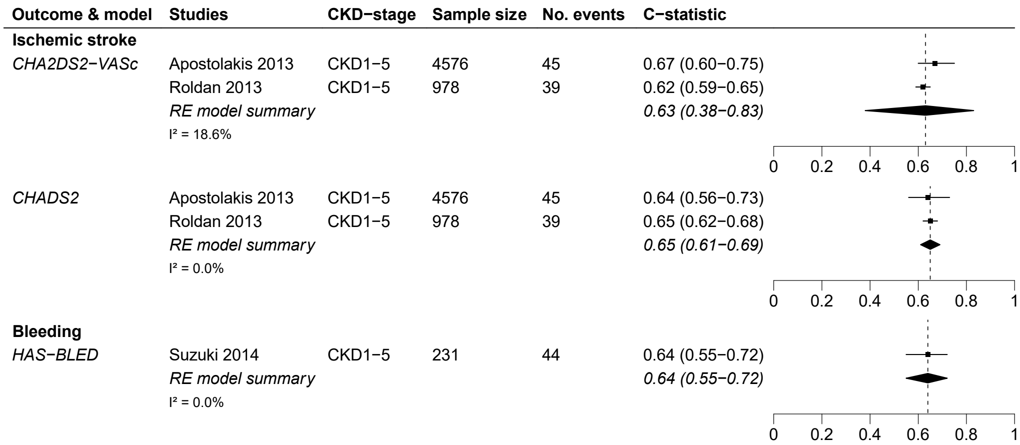


a)

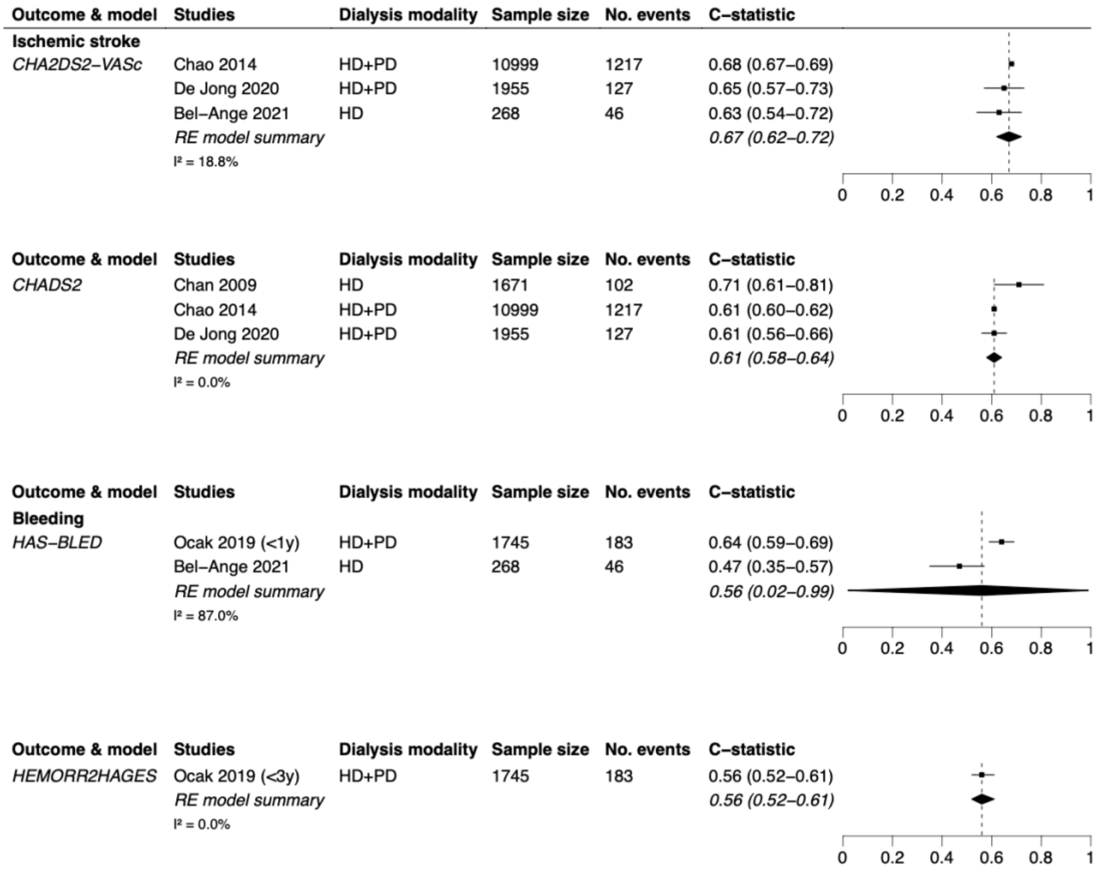


b)

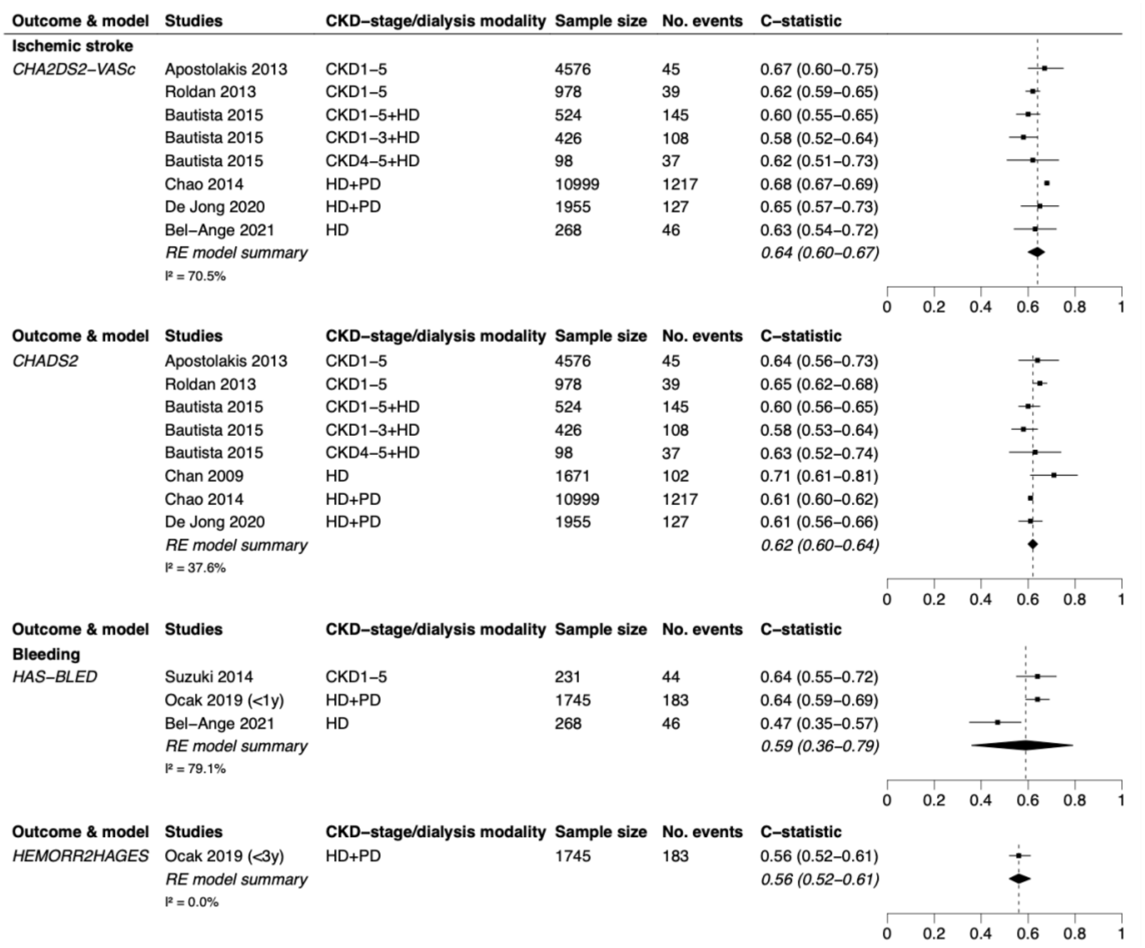
Outcome & model | Studies | CKD–stage/dialysis modality | Sample size | No. events | C–statistic
Ischemic stroke
CHA2DS2–VASc
Apostolakis 2013 | CKD1–5 | 4576 | 45 | 0.67 (0.60–0.75)
Roldan 2013 | CKD1–5 | 978 | 39 | 0.62 (0.59–0.65)
Bautista 2015 | CKD1–5+HD | 524 | 145 | 0.60 (0.55–0.65)
Bautista 2015 | CKD1–3+HD | 426 | 108 | 0.58 (0.52–0.64)
Bautista 2015 | CKD4–5+HD | 98 | 37 | 0.62 (0.51–0.73)
Chao 2014 | HD+PD | 10999 | 1217 | 0.68 (0.67–0.69)
De Jong 2020 | HD+PD | 1955 | 127 | 0.65 (0.57–0.73)
Bel–Ange 2021 | HD | 268 | 46 | 0.63 (0.54–0.72)
RE model summary | 0.64 (0.60–0.67)
I² = 70.5%
0 0.2 0.4 0.6 0.8 1
Outcome & model | Studies | CKD–stage/dialysis modality | Sample size | No. events | C–statistic
CHADS2
Apostolakis 2013 | CKD1–5 | 4576 | 45 | 0.64 (0.56–0.73)
Roldan 2013 | CKD1–5 | 978 | 39 | 0.65 (0.62–0.68)
Bautista 2015 | CKD1–5+HD | 524 | 145 | 0.60 (0.56–0.65)
Bautista 2015 | CKD1–3+HD | 426 | 108 | 0.58 (0.53–0.64)
Bautista 2015 | CKD4–5+HD | 98 | 37 | 0.63 (0.52–0.74)
Chan 2009 | HD | 1671 | 102 | 0.71 (0.61–0.81)
Chao 2014 | HD+PD | 10999 | 1217 | 0.61 (0.60–0.62)
De Jong 2020 | HD+PD | 1955 | 127 | 0.61 (0.56–0.66)
RE model summary | 0.62 (0.60–0.64)
I² = 37.6%
0 0.2 0.4 0.6 0.8 1
Outcome & model | Studies | CKD–stage/dialysis modality | Sample size | No. events | C–statistic
Bleeding
HAS–BLED
Suzuki 2014 | CKD1–5 | 231 | 44 | 0.64 (0.55–0.72)
Ocak 2019 (<1y) | HD+PD | 1745 | 183 | 0.64 (0.59–0.69)
Bel–Ange 2021 | HD | 268 | 46 | 0.47 (0.35–0.57)
RE model summary | 0.59 (0.36–0.79)
I² = 79.1%
0 0.2 0.4 0.6 0.8 1
Outcome & model | Studies | CKD–stage/dialysis modality | Sample size | No. events | C–statistic
HEMORR2HAGES
Ocak 2019 (<3y) | HD+PD | 1745 | 183 | 0.56 (0.52–0.61)
RE model summary | 0.56 (0.52–0.61)
I² = 0.0%
0 0.2 0.4 0.6 0.8 1

c)

**Figure S5: Forest plots sensitivity analysis 5**

a) Forest plots for CKD patients only, b) forest plots for dialysis patients only and c) forest plots for CKD and dialysis patients combined.

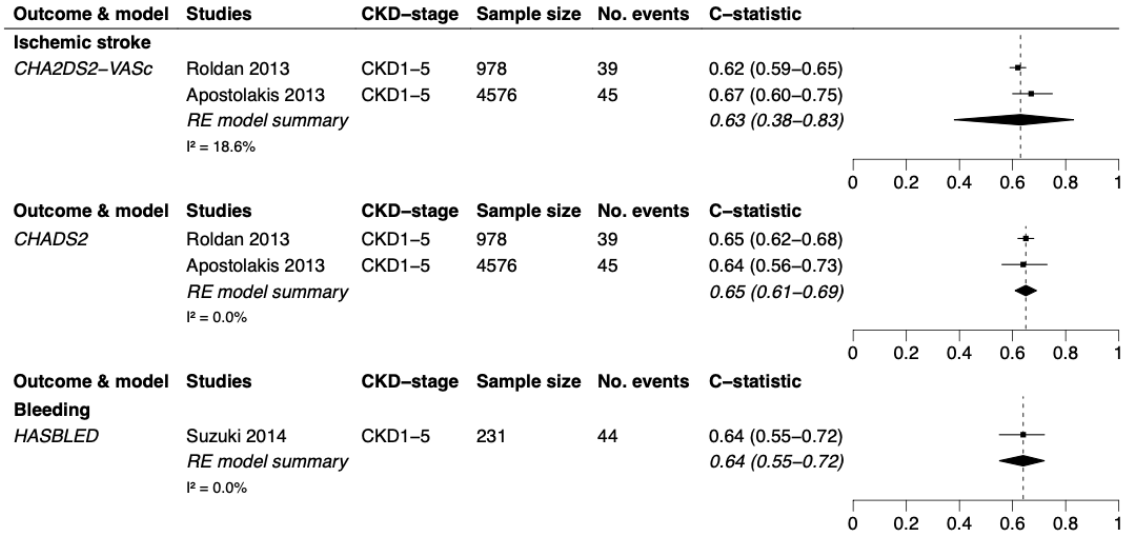


a)

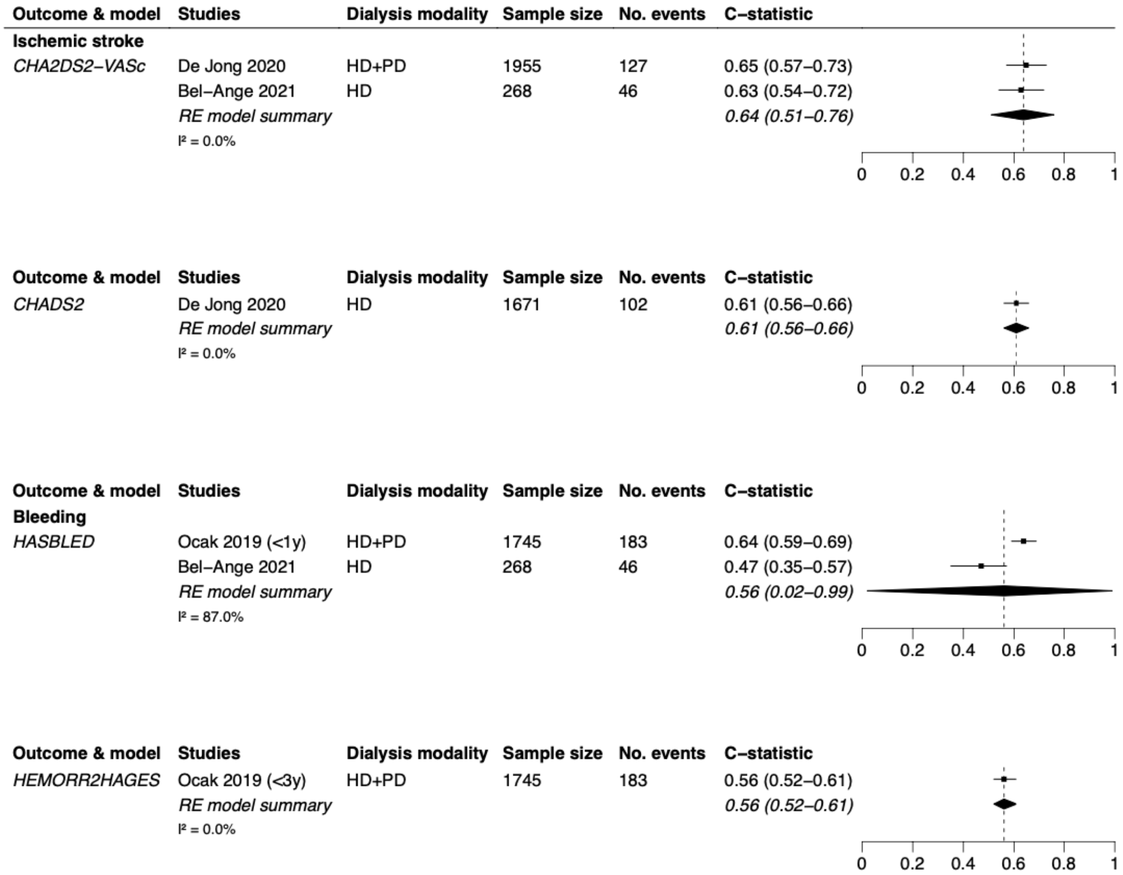


b)

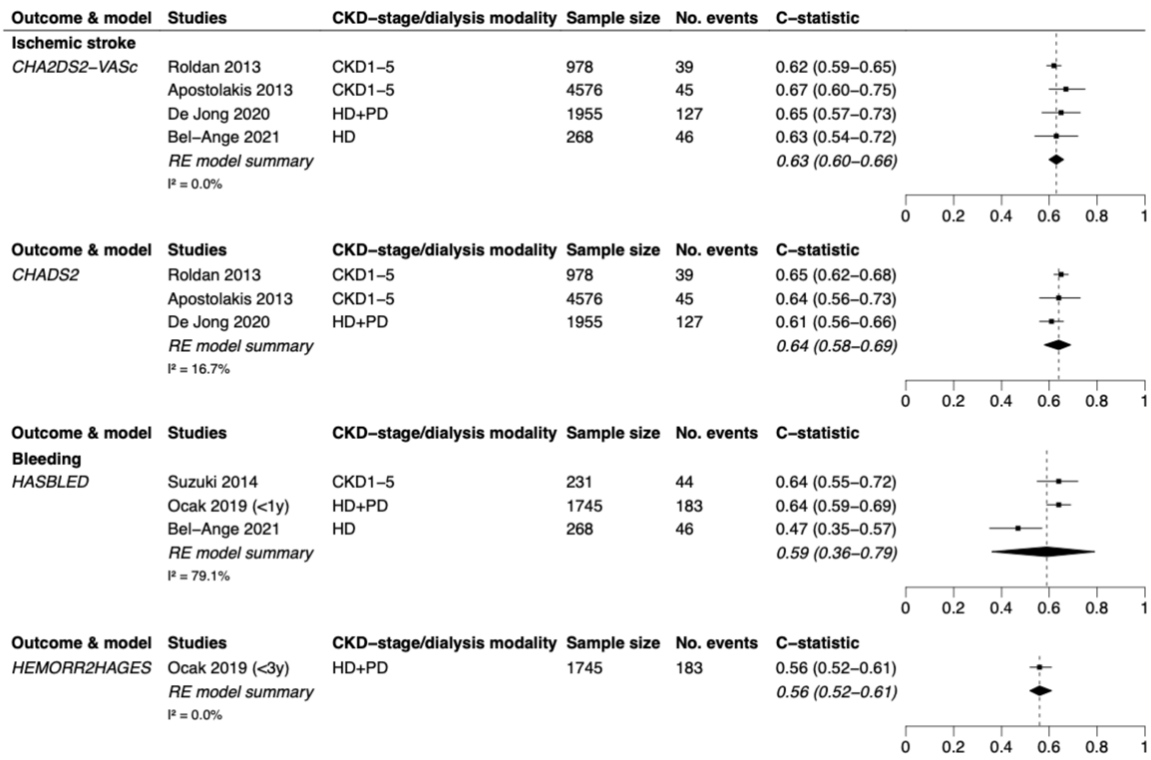
Outcome & model | Studies | CKD–stage/dialysis modality | Sample size | No. events | C–statistic
Ischemic stroke
CHA2DS2–VASc
Roldan 2013 | CKD1–5 | 978 | 39 | 0.62 (0.59–0.65)
Apostolakis 2013 | CKD1–5 | 4576 | 45 | 0.67 (0.60–0.75)
De Jong 2020 | HD+PD | 1955 | 127 | 0.65 (0.57–0.73)
Bel–Ange 2021 | HD | 268 | 46 | 0.63 (0.54–0.72)
RE model summary | 0.63 (0.60–0.66)
I² = 0.0%
0 0.2 0.4 0.6 0.8 1
Outcome & model | Studies | CKD–stage/dialysis modality | Sample size | No. events | C–statistic
CHADS2
Roldan 2013 | CKD1–5 | 978 | 39 | 0.65 (0.62–0.68)
Apostolakis 2013 | CKD1–5 | 4576 | 45 | 0.64 (0.56–0.73)
De Jong 2020 | HD+PD | 1955 | 127 | 0.61 (0.56–0.66)
RE model summary | 0.64 (0.58–0.69)
I² = 16.7%
0 0.2 0.4 0.6 0.8 1
Outcome & model | Studies | CKD–stage/dialysis modality | Sample size | No. events | C–statistic
Bleeding
HASBLED
Suzuki 2014 | CKD1–5 | 231 | 44 | 0.64 (0.55–0.72)
Ocak 2019 (<1y) | HD+PD | 1745 | 183 | 0.64 (0.59–0.69)
Bel–Ange 2021 | HD | 268 | 46 | 0.47 (0.35–0.57)
RE model summary | 0.59 (0.36–0.79)
I² = 79.1%
0 0.2 0.4 0.6 0.8 1
Outcome & model | Studies | CKD–stage/dialysis modality | Sample size | No. events | C–statistic
HEMORR2HAGES
Ocak 2019 (<3y) | HD+PD | 1745 | 183 | 0.56 (0.52–0.61)
RE model summary | 0.56 (0.52–0.61)
I² = 0.0%
0 0.2 0.4 0.6 0.8 1

c)

**Figure S6: Forest plots sensitivity analysis 6**

a) Forest plots for CKD patients only, b) forest plots for dialysis patients only and c) forest plots for CKD and dialysis patients combined.

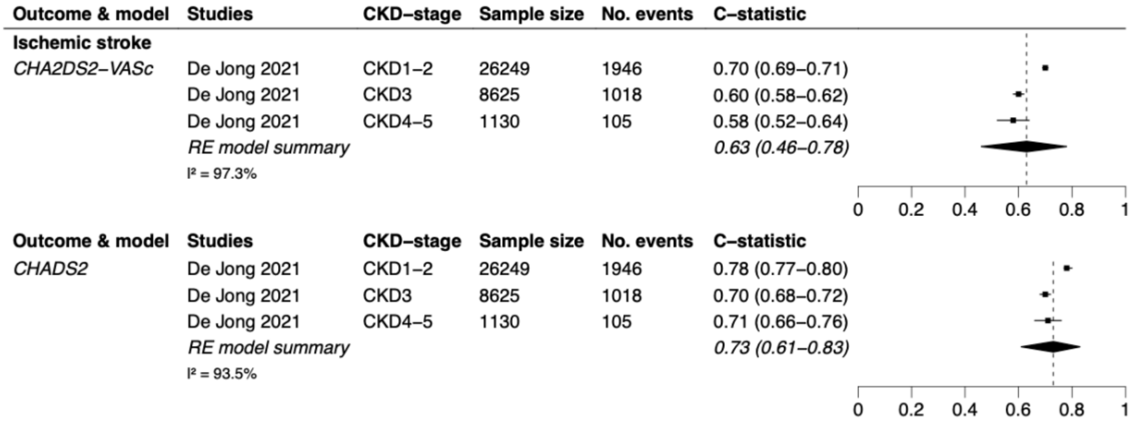


a)

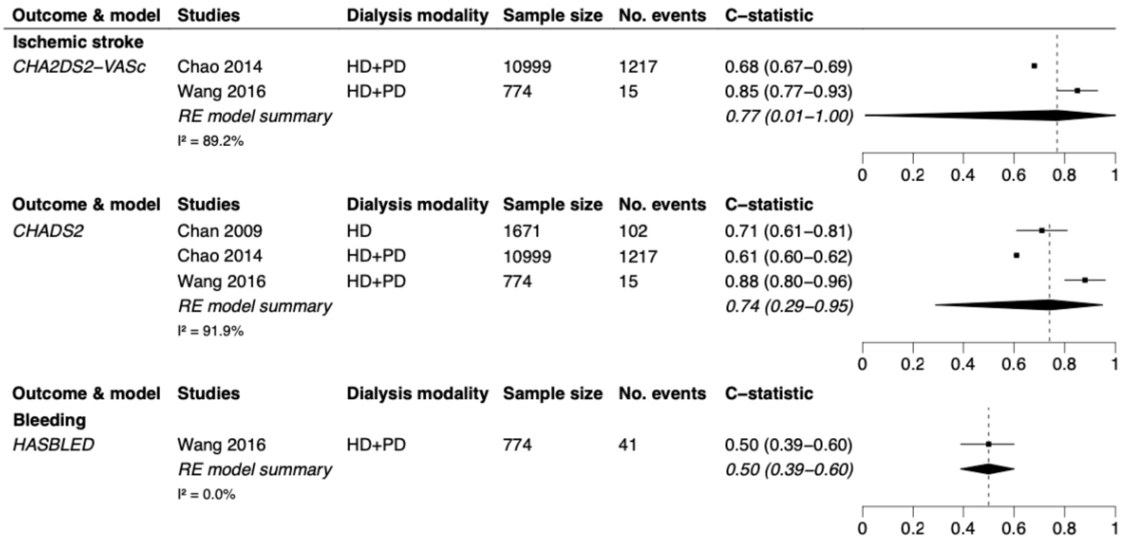


b)

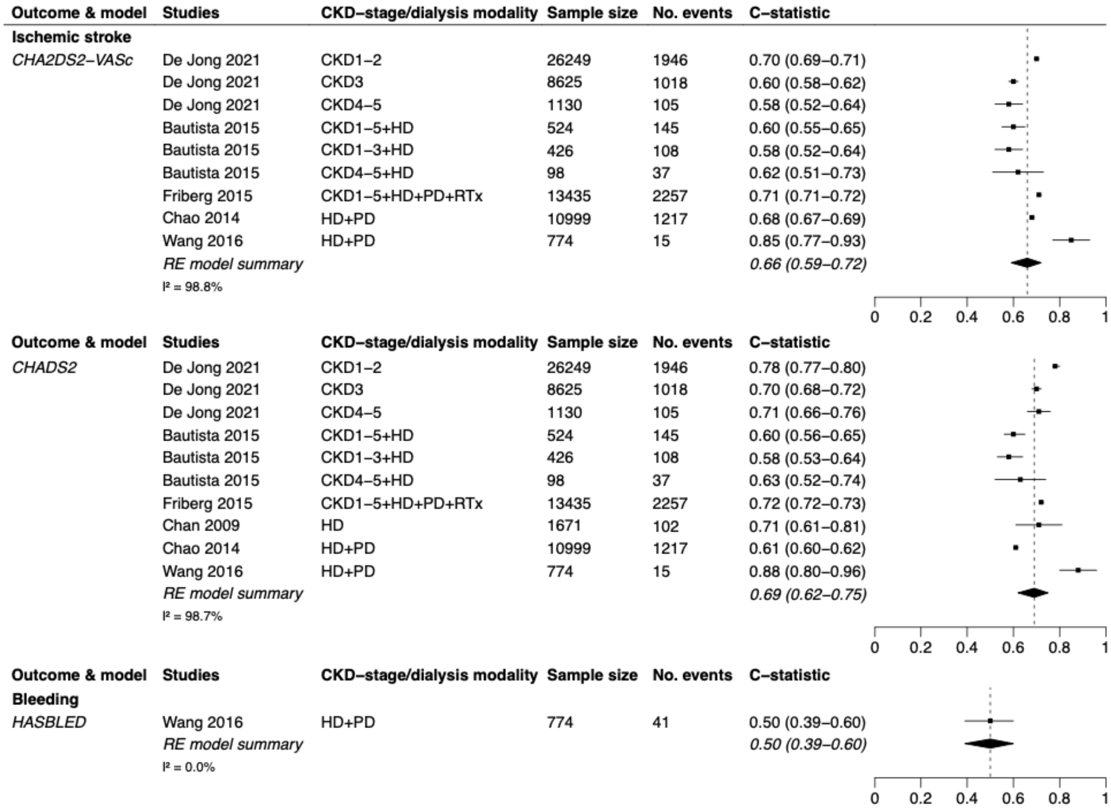
Outcome & model | Studies | CKD−stage/dialysis modality | Sample size | No. events | C−statistic
Ischemic stroke
CHA2DS2−VASc | De Jong 2021 | CKD1−2 | 26249 | 1946 | 0.70 (0.69−0.71)
De Jong 2021 | CKD3 | 8625 | 1018 | 0.60 (0.58−0.62)
De Jong 2021 | CKD4−5 | 1130 | 105 | 0.58 (0.52−0.64)
Bautista 2015 | CKD1−5+HD | 524 | 145 | 0.60 (0.55−0.65)
Bautista 2015 | CKD1−3+HD | 426 | 108 | 0.58 (0.52−0.64)
Bautista 2015 | CKD4−5+HD | 98 | 37 | 0.62 (0.51−0.73)
Friberg 2015 | CKD1−5+HD+PD+RTx | 13435 | 2257 | 0.71 (0.71−0.72)
Chao 2014 | HD+PD | 10999 | 1217 | 0.68 (0.67−0.69)
Wang 2016 | HD+PD | 774 | 15 | 0.85 (0.77−0.93)
RE model summary | 0.66 (0.59−0.72)
I² = 98.8%
0 0.2 0.4 0.6 0.8 1
Outcome & model | Studies | CKD−stage/dialysis modality | Sample size | No. events | C−statistic
CHADS2 | De Jong 2021 | CKD1−2 | 26249 | 1946 | 0.78 (0.77−0.80)
De Jong 2021 | CKD3 | 8625 | 1018 | 0.70 (0.68−0.72)
De Jong 2021 | CKD4−5 | 1130 | 105 | 0.71 (0.66−0.76)
Bautista 2015 | CKD1−5+HD | 524 | 145 | 0.60 (0.56−0.65)
Bautista 2015 | CKD1−3+HD | 426 | 108 | 0.58 (0.53−0.64)
Bautista 2015 | CKD4−5+HD | 98 | 37 | 0.63 (0.52−0.74)
Friberg 2015 | CKD1−5+HD+PD+RTx | 13435 | 2257 | 0.72 (0.72−0.73)
Chan 2009 | HD | 1671 | 102 | 0.71 (0.61−0.81)
Chao 2014 | HD+PD | 10999 | 1217 | 0.61 (0.60−0.62)
Wang 2016 | HD+PD | 774 | 15 | 0.88 (0.80−0.96)
RE model summary | 0.69 (0.62−0.75)
I² = 98.7%
0 0.2 0.4 0.6 0.8 1
Outcome & model | Studies | CKD−stage/dialysis modality | Sample size | No. events | C−statistic
Bleeding
HASBLED | Wang 2016 | HD+PD | 774 | 41 | 0.50 (0.39−0.60)
RE model summary | 0.50 (0.39−0.60)
I² = 0.0%
0 0.2 0.4 0.6 0.8 1

c)

**Interpretation of sensitivity analyses**

*Sensitivity analysis 1*

The first sensitivity analysis, including only patients with prevalent CKD and dialysis, showed a pooled c-statistic of 0.66 (95% confidence interval 0.60-0.71) for the $CHA_2DS_2$-VASc and 0.69 (0.62-0.76) for the $CHADS_2$ regarding IS prediction in this combined group. In these patients, the HAS-BLED performed similarly to the $HEMORR_2HAGES$ regarding bleeding prediction, with c-statistics of 0.57 (0.42-0.71) vs. 0.56 (0.52-0.61) respectively (**Supplementary Figure S1**). Both results are in accordance with the results of the combined group in our main analysis.

*Sensitivity analysis 2*

The second sensitivity analysis, including only patients with unclearly defined CKD or dialysis, showed that in this combined group the $CHA_2DS_2$-VASc performed similarly to the $CHADS_2$ regarding IS prediction, with c-statistics of 0.63 (0.53-0.73) vs. 0.64 (0.53-0.74) respectively (**Supplementary Figure S2**). Forest plots and pooled c-statistics were not calculated for bleeding risk due to the absence of validations of the HAS-BLED and $HEMORR_2HAGES$ in this population.

*Sensitivity analysis 3*

The third sensitivity analysis, including only patients with incident AF, showed that for CKD patients only the $CHADS_2$ showed a higher c-statistic than the $CHA_2DS_2$-VASc regarding IS prediction, with c-statistics of 0.73 (0.61-0.83) vs. 0.63 (0.46-0.78) respectively (**Supplementary Figure S3a**). For dialysis patients only, the discrimination results of IS prediction showed a pooled c-statstic of 0.85 (0.77-0.93) for the $CHA_2DS_2$-VASc and 0.88 (0.80-0.96) for the $CHADS_2$ (**Supplementary Figure S3b**). For CKD and dialysis patients combined, the $CHADS_2$ showed a slightly higher discriminative ability than the $CHA_2DS_2$-VASc regarding IS prediction, with c-statistics of 0.74 (0.66-0.81) vs. 0.69 (0.54-0.80)

respectively (**Supplementary Figure S3c**), corresponding to the results of our main analysis. Forest plots and pooled c-statistics were not calculated for bleeding risk due to the absence of validations of the $HEMORR_2HAGES$ in this population.

*Sensitivity analysis 4*

The fourth sensitivity analysis, including only patients with non-incident and unclear type AF, showed that for CKD patients only the $CHA_2DS_2$-VASc and $CHADS_2$ performed similarly regarding IS prediction, with c-statistics of 0.63 (0.38-0.83) vs. 0.65 (0.61-0.69) respectively (**Supplementary Figure S4a**). Forest plots and pooled c-statistics were not calculated for bleeding risk due to the absence of validations of the $HEMORR_2HAGES$ in this population. For dialysis patients only, the $CHA_2DS_2$-VASc showed a slightly higher c-statistic than the $CHADS_2$ regarding IS prediction, with c-statistics of 0.67 (0.62-0.72) vs. 0.61 (0.58-0.64) respectively, whereas our main analysis showed a similar performance of these prediction models in dialysis patients. In these patients, the HAS-BLED performed similarly to the $HEMORR_2HAGES$ regarding bleeding prediction, with c-statistics of 0.56 (0.02-0.99) vs. 0.56 (0.52-0.61) respectively (**Supplementary Figure S4b**). For CKD and dialysis patients combined, the the $CHA_2DS_2$-VASc and $CHADS_2$ performed similarly regarding IS prediction, with c-statistics of 0.64 (0.60-0.67) vs. 0.62 (0.60-0.64) respectively. In these patients, the HAS-BLED performed similarly to the $HEMORR_2HAGES$ regarding bleeding prediction, with c-statistics of 0.59 (0.36-0.79) vs. 0.56 (0.52-0.61) respectively (**Supplementary Figure S4c**).

*Sensitivity analysis 5*

The fifth sensitivity analysis, including only studies with a prospective study design, showed that for CKD patients only the $CHA_2DS_2$-VASc and $CHADS_2$ performed similarly regarding IS prediction, with c-statistics of 0.63 (0.38-0.83) vs. 0.65 (0.61-0.69) respectively (**Supplementary Figure S5a**) whereas our

main analysis showed a slightly better performance of the $CHADS_2$ compared to the $CHA_2DS_2$-VASc in CKD patients. Forest plots and pooled c-statistics were not calculated for bleeding risk due to the absence of validations of the $HEMORR_2HAGES$ in this population. For dialysis patients only, the $CHA_2DS_2$-VASc performed similarly to the $CHADS_2$ regarding IS prediction, with c-statistics of 0.64 (0.51-0.76) vs. 0.61 (0.56-0.66) respectively. In these patients, the HAS-BLED performed similarly to the $HEMORR_2HAGES$ regarding bleeding prediction, with c-statistics of 0.56 (0.02-0.99) vs. 0.56 (0.52-0.61) respectively (**Supplementary Figure S5b**). For CKD and dialysis patients combined, the the $CHA_2DS_2$-VASc and $CHADS_2$ performed similarly regarding IS prediction, with c-statistics of 0.63 (0.60-0.66) vs. 0.64 (0.58-0.69) respectively. In these patients, the HAS-BLED performed similarly to the $HEMORR_2HAGES$ regarding bleeding prediction, with c-statistics of 0.59 (0.36-0.79) vs. 0.56 (0.52-0.61) respectively (**Supplementary Figure S5c**). The results for dialysis patients and CKD and dialysis patients combined are in accordance with the results of our main analysis.

*Sensitivity analysis 6*

The sixth sensitivity analysis, including only studies with a retrospective study design, showed that for CKD patients only the $CHADS_2$ showed a higher c-statistic than the $CHA_2DS_2$-VASc regarding IS prediction, with c-statistics of 0.73 (0.61-0.83) vs. 0.63 (0.46-0.78) respectively (**Supplementary Figure S6a**). Forest plots and pooled c-statistics were not calculated for bleeding risk due to the absence of validations of the HAS-BLED and $HEMORR_2HAGES$ in this population. For dialysis patients only, the $CHA_2DS_2$-VASc showed a similar performance to the $CHADS_2$ regarding IS prediction, with c-statistics of 0.77 (0.01-1.00) vs. 0.74 (0.29-0.95) respectively (**Supplementary Figure S6b**). Forest plots and pooled c-statistics were not calculated for bleeding risk due to the absence of validations of the $HEMORR_2HAGES$ in this population. For CKD and dialysis patients combined, the the $CHA_2DS_2$-VASc and $CHADS_2$ performed similarly regarding IS prediction, with c-statistics of 0.66 (0.59-0.72) vs. 0.69

(0.62-0.75) respectively (**Supplementary Figure S6c**). In this population, forest plots and pooled c-statistics were not calculated for bleeding risk due to the absence of validations of the $HEMORR_2HAGES$. All other results are in accordance with our main analysis.

## SECTION C: EVENT RATES (EVR), CUMULATIVE INCIDENCE AND CALIBRATION

**Table S3: EVR stroke by CHA2DS2-VASc**

| Score/study | Chao[35] | Genovesi[77] | Lee[48] | See[50] | Shih[44] | Sy[51] |
|---|---|---|---|---|---|---|
| 0 | 2.1 | 0 | 1.32 | 2.44 | 1.82 | 1.5 |
| 1 | 2.4 | | 1.19 | | 1.15 | |
| 2 | 2.8 | 2.5 | 1.22 | | 1.97 | |
| 3 | 4.2 | | 0.45 | | 2.81 | |
| 4 | 6.4 | | 2.76 | 5.59 | 2.56 | 1.5 |
| 5 | 7.8 | 6.2 | 4.01 | | 3.84 | 2.5 |
| 6 | 10 | | 13.20 | | 4.30 | 3.0 |
| 7 | 11 | | 4.58 | | 5.02 | 3.5 |
| 8 | 12.4 | | 2.87 | | 7.25 | 4.5 |
| 9 | 13.1 | | 28.65 | | 8.66 | |

**Table S4: EVR stroke by CHADS2**

| Score/ study | Chao[35] | Hasegawa[43] | Shah[49] | Shih[44] | Sood[45] | Wakasugi[46] | Wizemann[47] |
|---|---|---|---|---|---|---|---|
| 0 | 2.0 | 5.3 | 1.99 | 1.60 | 1 | N.A. | 0.5 |
| 1 | 3.5 | 4.4 | 2.35 | 2.70 | 2 | 0 | 2.1 |
| 2 | 4.1 | 8.2 | 3.55 | 2.22 | 4 | 5.3 | 1.9 |
| 3 | 7.1 | | | 4.01 | | 26.1 | 3.9 |
| 4 | 9.9 | | | 4.23 | | 10.3 | 6.0 |
| 5 | 12 | | | 5.97 | | 72.0 | 6.5 |
| 6 | 11.2 | | | 7.37 | | N.A. | 12.7 |

**Table S5: EVR bleeding by HAS-BLED**

| Score/study | Genovesi[77] | See[50] | Shah[49] | Sy[51] |
|---|---|---|---|---|
| 0 | 0 | 4.26 | N.A. | 7 |
| 1 | | | 8.00 | |
| 2 | 3.8 | | | |
| 3 | | 5.53 | 9.08 | 10 |
| 4 | 16 | | | 9 |
| 5 | | | | 11 |
| 6 | | | | 13 |
| 7 | | | | |
| 8 | | | | |
| 9 | | | | N.A. |

CI = $1 - e^{-IR \times D}$
IR = EVR : 100
D = 1 year for the $CHA_2DS_2$-VASc and HAS-BLED and 2.7 years (i.e., censoring after 1000 days) for $CHADS_2$ and $HEMORR_2HAGES$
N.A. = not available

**Table S6: Observed cumulative incidence of stroke and bleeding by the CHADS$_2$, CHA$_2$DS$_2$-VASc, HAS-BLED and HEMORR$_2$HAGES in patients on dialysis**

| | | HD | | | | | | | | PD | | HD & PD | | | Unclear dialysis modality | |
|---|---|---|---|---|---|---|---|---|---|---|---|---|---|---|---|---|
| **Score (prediction horizon)** | **Predicted** | **Bel-Ange[42]** | **Genovesi[77]** | **Hasegawa[43]** | **Schamroth Pravda[52]** | **Shih[44]** | **Sood[45]** | **Wakasugi[46]** | **Wizemann[47]** | **Chan[34]** | **Lee[48]** | **Shah[49]** | **Chao[35]** | **Ocak[31]** | **See[50]** | **Wetmore[81]** |
| **CHADS$_2$ (=1000 days)** | | | | | | | | | | | | | | | | |
| 0 | 0.019 | | | 0.13[1] | | 0.04[1] | 0.027[1] | N.A. | 0.01[1] | | | 0.05[1] | 0.05[1] | | | 0.06[3] |
| 1 | 0.028 | | | 0.11[1] | | 0.07[1] | 0.053[1] | 0 | 0.06[1] | | | 0.06[1] | 0.09[1] | | | |
| 2 | 0.04 | | | 0.20[1] | | 0.06[1] | 0.102[1] | 0.13[1] | 0.05[1] | | | 0.09[1] | 0.10[1] | | | 0.11[3] |
| 3 | 0.059 | | | | | 0.10[1] | | 0.51[1] | 0.10[1] | | | | 0.17[1] | | | 0.13[3] |
| 4 | 0.085 | | | | | 0.11[1] | | 0.24[1] | 0.15[1] | | | | 0.23[1] | | | |
| 5 | 0.125 | | | | | 0.15[1] | | 0.86[1] | 0.16[1] | | | | 0.28[1] | | | |
| 6 | 0.182 | | | | | 0.18[1] | | N.A. | 0.29[1] | | | | 0.26[1] | | | |
| **CHA$_2$DS$_2$-VASc (=1 y)** | | | | | | | | | | | | | | | | |
| 0 | 0.002 | 0[3] | 0[1] | | 0.017[2] | 0.02[1] | | | | 0[3] | 0.01[1] | | 0.02[1] | | 0.02[1] | |
| 1 | 0.006 | | | | | 0.01[1] | | | | | 0.01[1] | | 0.02[1] | | | |
| 2 | 0.022 | | 0.02[1] | | | 0.02[1] | | | | 0.11[3] | 0.01[1] | | 0.03[1] | | | |
| 3 | 0.032 | 0.05[3] | | | | 0.03[1] | | | | | 0.004[1] | | 0.04[1] | | | |
| 4 | 0.048 | | | | 0.041[2] | 0.03[1] | | | | | 0.03[1] | | 0.06[1] | | 0.05[1] | |
| 5 | 0.072 | | 0.06[1] | | | 0.04[1] | | | | | 0.04[1] | | 0.08[1] | | | |
| 6 | 0.097 | 0.15[3] | | | 0.072[2] | 0.04[1] | | | | | 0.12[1] | | 0.10[1] | | | |
| 7 | 0.112 | | | | | 0.05[1] | | | | | 0.04[1] | | 0.10[1] | | | |
| 8 | 0.108 | | | | | 0.07[1] | | | | | 0.03[1] | | 0.12[1] | | | |
| 9 | 0.122 | | | | | 0.08[1] | | | | | 0.20[1] | | 0.12[1] | | | |
| **HAS-BLED (=1 y)** | | | | | | | | | | | | | | | | |
| 0 | 0.009 | 0.23[3] | 0[1] | | | | | | | | | N.A. | | 0.02[3] | 0.04[1] | |
| 1 | 0.034 | | | | | | | | | | | 0.08[1] | | | | |
| 2 | 0.041 | | 0.04[1] | | | | | | | | | | | 0.08[3] | | |
| 3 | 0.058 | 0.06[3] | | | | | | | | | | 0.09[1] | | 0.1[3] | 0.05[1] | |
| 4 | 0.089 | | 0.15[1] | | | | | | | | | | | | | |

| | | | | | | | | | | | | | | | | |
|---|---|---|---|---|---|---|---|---|---|---|---|---|---|---|---|---|
| 5 | 0.091 | | | | | | | | | | | | | | | |
| 6 | > 0.1 | 0.05[3] | | | | | | | | | | | | | | |
| 7 | > 0.1 | | | | | | | | | | | | | | | |
| 8 | > 0.1 | | | | | | | | | | | | | | | |
| 9 | > 0.1 | | | | | | | | | | | | | | | |
| **$HEMORR_2HAGES$ (=1000 days)** | | | | | | | | | | | | | | | | |
| 0 | 0.019 | | | | | | | | | | | | | 0.07[3] | | |
| 1 | 0.025 | | | | | | | | | | | | | | | |
| 2 | 0.053 | | | | | | | | | | | | | 0.13[3] | | |
| 3 | 0.084 | | | | | | | | | | | | | | | |
| 4 | 0.104 | | | | | | | | | | | | | 0.19[3] | | |
| 5 | 0.123 | | | | | | | | | | | | | | | |
| 6 | 0.123 | | | | | | | | | | | | | | | |
| 7 | 0.123 | | | | | | | | | | | | | | | |
| 8 | 0.123 | | | | | | | | | | | | | | | |
| 9 | 0.123 | | | | | | | | | | | | | | | |
| 10 | 0.123 | | | | | | | | | | | | | | | |
| 11 | 0.123 | | | | | | | | | | | | | | | |
| 12 | 0.123 | | | | | | | | | | | | | | | |

[1] reported as EVR (event rate, and if need recalculated to number of events / 100 person years (PY)), and subsequently recalculated to cumulative incidence at the prediction horizon as defined in the development study (i.e., one year for the $CHA_2DS_2$-VASc, one year for the HAS-BLED, and two years and eight months (i.e., censoring after 1000 days) for the $CHADS_2$ and $HEMORR_2HAGES$). Recalculation methods and original EVR's are given in **Supplementary Table S2-4**.

[2] reported as cumulative incidence at the prediction timeframe of the development studies

[3] reported as cumulative incidence at a different prediction timeframe than the development studies, but approximated from the Kaplan-Meier graph at the relevant prediction timeframe

N.A. not available

**Table S7: Observed cumulative incidence of stroke and bleeding by the $CHADS_2$, $CHA_2DS_2$-VASc, HAS-BLED and $HEMORR_2HAGES$ in patients with chronic kidney disease**

| | **Chronic kidney disease** | |
|---|---|---|
| **Score (prediction horizon)** | **Predicted** | **Sy[51]** |
| **CHADS2 (= 1000 days)** | | |
| 0 | 0.019 | |
| 1 | 0.028 | |
| 2 | 0.04 | |
| 3 | 0.059 | |
| 4 | 0.085 | |
| 5 | 0.125 | |
| 6 | 0.182 | |
| **CHA2DS2-VASc (=1 y)** | | |
| 0 | 0.002 | 0.015[1] |
| 1 | 0.006 | |
| 2 | 0.022 | |
| 3 | 0.032 | |
| 4 | 0.048 | 0.015[1] |
| 5 | 0.072 | 0.025[1] |
| 6 | 0.097 | 0.030[1] |
| 7 | 0.112 | 0.034[1] |
| 8 | 0.108 | 0.044[1] |
| 9 | 0.122 | |
| **HAS-BLED (=1 y)** | | |
| 0 | 0.009 | 0.068[1] |
| 1 | 0.034 | |
| 2 | 0.041 | |
| 3 | 0.058 | 0.095[1] |
| 4 | 0.089 | 0.086[1] |
| 5 | 0.091 | 0.104[1] |
| 6 | > 0.1 | 0.122[1] |
| 7 | > 0.1 | |
| 8 | > 0.1 | |
| 9 | > 0.1 | N.A. |
| **HEMORR2 HAGES (=1000 days)** | | |
| 0 | 0.019 | |
| 1 | 0.025 | |
| 2 | 0.053 | |
| 3 | 0.084 | |
| 4 | 0.104 | |
| 5 | 0.123 | |

| | | |
|---|---|---|
| 6 | 0.123 | |
| 7 | 0.123 | |
| 8 | 0.123 | |
| 9 | 0.123 | |
| 10 | 0.123 | |
| 11 | 0.123 | |
| 12 | 0.123 | |

[1] reported as EVR (event rate, and if need recalculated to number of events / 100 person years (PY)), and subsequently recalculated to cumulative incidence at the prediction horizon as defined in the development study (i.e., one year for the $CHA_2DS_2$-VASc, one year for the HAS-BLED, and two years and eight months (i.e., censoring after 1000 days) for the $CHADS_2$ and $HEMORR_2HAGES$). Recalculation methods are given in. Recalculation methods and original EVR is reported in the **Supplement Table S2-4**.
N.A. not available

**Table S8: Stroke and bleeding outcomes per anticoagulant class**

| Study | Outcome | Anticoagulant | n/N | Incidence per 1000 patients/ year | Cumulative incidence rate (n/100 person-years) | Hazard ratio |
|---|---|---|---|---|---|---|
| Eikelboom[67] | IS | Apixaban | 51/2774 | NA | NA | NA |
| | | Aspirin | 111/2751 | NA | NA | NA |
| | Bleeding | Apixaban | 43/2774 | NA | NA | NA |
| | | Aspirin | 38/2751 | NA | NA | 1 (ref) |
| Calderon[66] | IS | NOAC | 1446/7244 | 399 | NA | NA |
| | | VKA | 1310/13543 | 64 | NA | NA |
| | | Aspirin | 5102/41991 | 133 | NA | NA |
| | Bleeding | NOAC | 233/14426 | 26 | NA | NA |
| | | VKA | 1152/42741 | 37 | NA | NA |
| | | Aspirin | 479/22278 | 30 | NA | NA |
| Kee[70] | IS | DOAC | 146/915 | NA | NA | NA |
| | | Warfarin | 147/970 | NA | NA | NA |
| | Bleeding | DOAC | 170/915 | NA | 5.58 | 0.64 |
| | | Warfarin | 313/970 | NA | 6.04 | 1 (ref) |
| Park[83] | IS | DOAC | 1/48 | NA | 0.9 | 0.61 |
| | | Warfarin | 3/114 | NA | 1.5 | 1 (ref) |
| | Bleeding | DOAC | 2/48 | NA | 1.8 | 0.23 |
| | | Warfarin | 16/114 | NA | 7.9 | 1 (ref) |
| Pokorney[79] | IS | Apixaban | 1/82 | NA | NA | NA |
| | | Warfarin | 2/72 | NA | NA | NA |
| | Bleeding | Apixaban | 21/82 | NA | NA | NA |
| | | Warfarin | 16/72 | NA | NA | NA |
| Reinecke[80] | IS | Apixaban | 0/48 | NA | NA | NA |
| | | Phenprocoumon | 1/49 | NA | NA | NA |
| | Bleeding | Apixaban | 5/48 | NA | NA | NA |
| | | Phenprocoumon | 6/49 | NA | NA | NA |
| Welander[84] | IS | DOAC | 28/1005 | NA | 2.2 | NA |
| | | Warfarin | 63/1448 | NA | 2.0 | NA |
| | Bleeding | DOAC | 77/1005 | NA | 6.3 | NA |
| | | Warfarin | 275/1448 | NA | 9.7 | NA |
| Wetmore[81] | IS | Warfarin | 424 | NA | 2.1 | 1 (ref) |
| | | Apixaban concordant | 52 | NA | 2.0 | 0.89 |
| | | Apixaban below label | 54 | NA | 1.9 | 0.85 |
| | Bleeding | Warfarin | 1226 | NA | 6.3 | 1 (ref) |
| | | Apixaban concordant | 127 | NA | 4.5 | 0.67 |
| | | Apixaban below label | 117 | NA | 4.7 | 0.68 |
| See[50] | IS | DOAC | NA | NA | 6.67 | 1.21 |
| | | Warfarin | NA | NA | 5.30 | 1 (ref) |
| | Bleeding | DOAC | NA | NA | 13.66 | 0.94 |
| | | Warfarin | NA | NA | 13.69 | 1 (ref) |
| Sood[45] | IS | OAC | NA | NA | NA | 1.51 |
| | | APA | NA | NA | NA | 1.79 |
| | | ASA | NA | NA | NA | 1.17 |
| | Bleeding | OAC | NA | NA | NA | 1.39 |
| | | APA | NA | NA | NA | 1.25 |
| | | ASA | NA | NA | NA | 1.13 |

Abreviations: n = number of events, N = total number of patients, NA = not applicable or not available, DOAC = direct oral anticoagulant, OAC = oral anticoagulant, APA = anti-platelet agent, ASA = acetylsalicyclic acid, NOAC = non-vitamin K antagonist oral anticoagulant, VKA = vitamin K antagonist, ref = reference.

**Figure S7: Calibration plots for the $CHA_2DS_2$-VASc in all dialysis types**

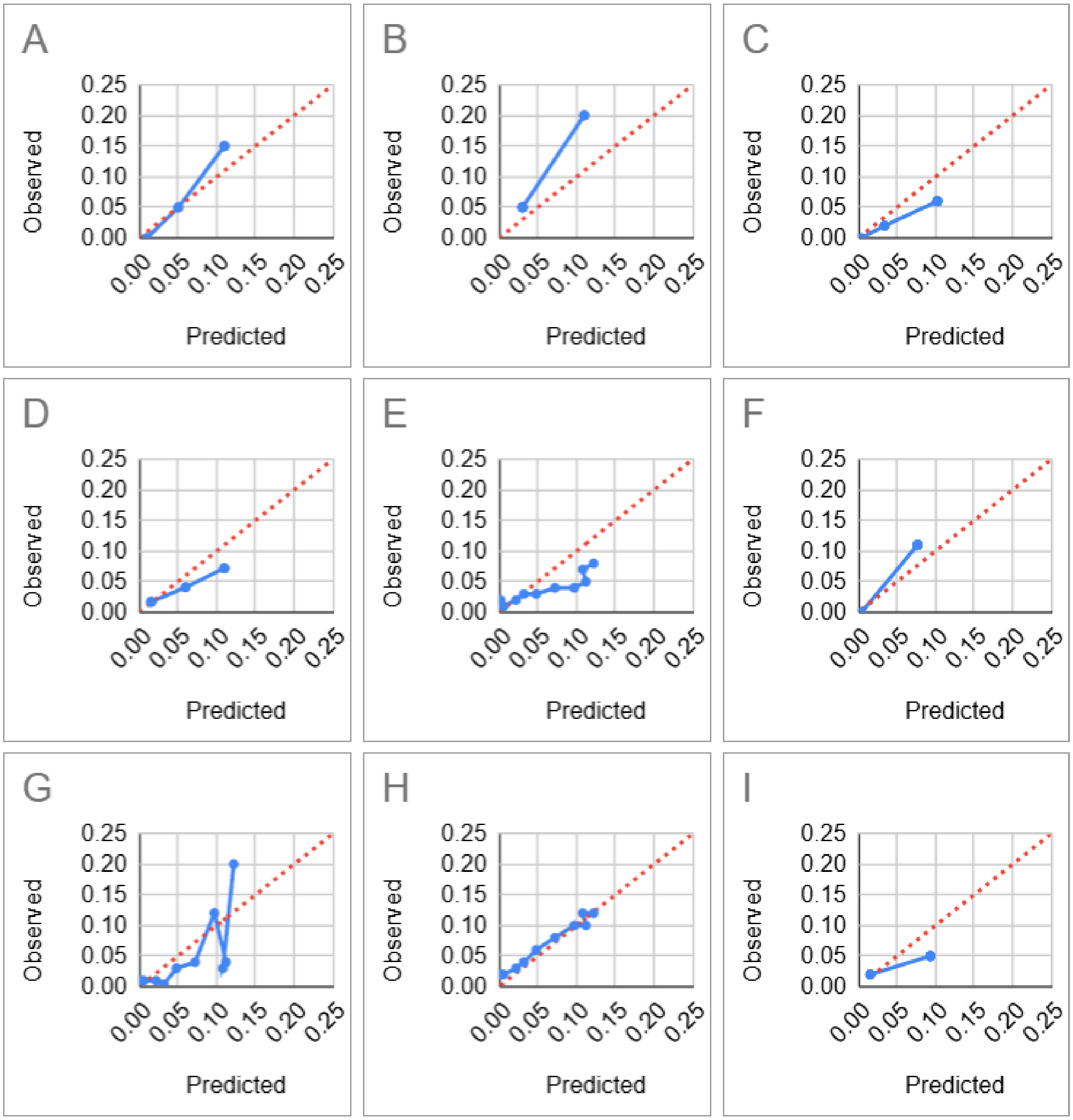


A. Bel-Ange 2021
B. Bel-Ange 2021
C. Genovesi 2017
D. Schamroth Pravda 2020
E. Shih 2016
F. Chan 2009
G. Lee 2020
H. Chao 2014
I. See 2021

**Figure S8: Calibration plots for the $CHADS_2$ in all dialysis types**

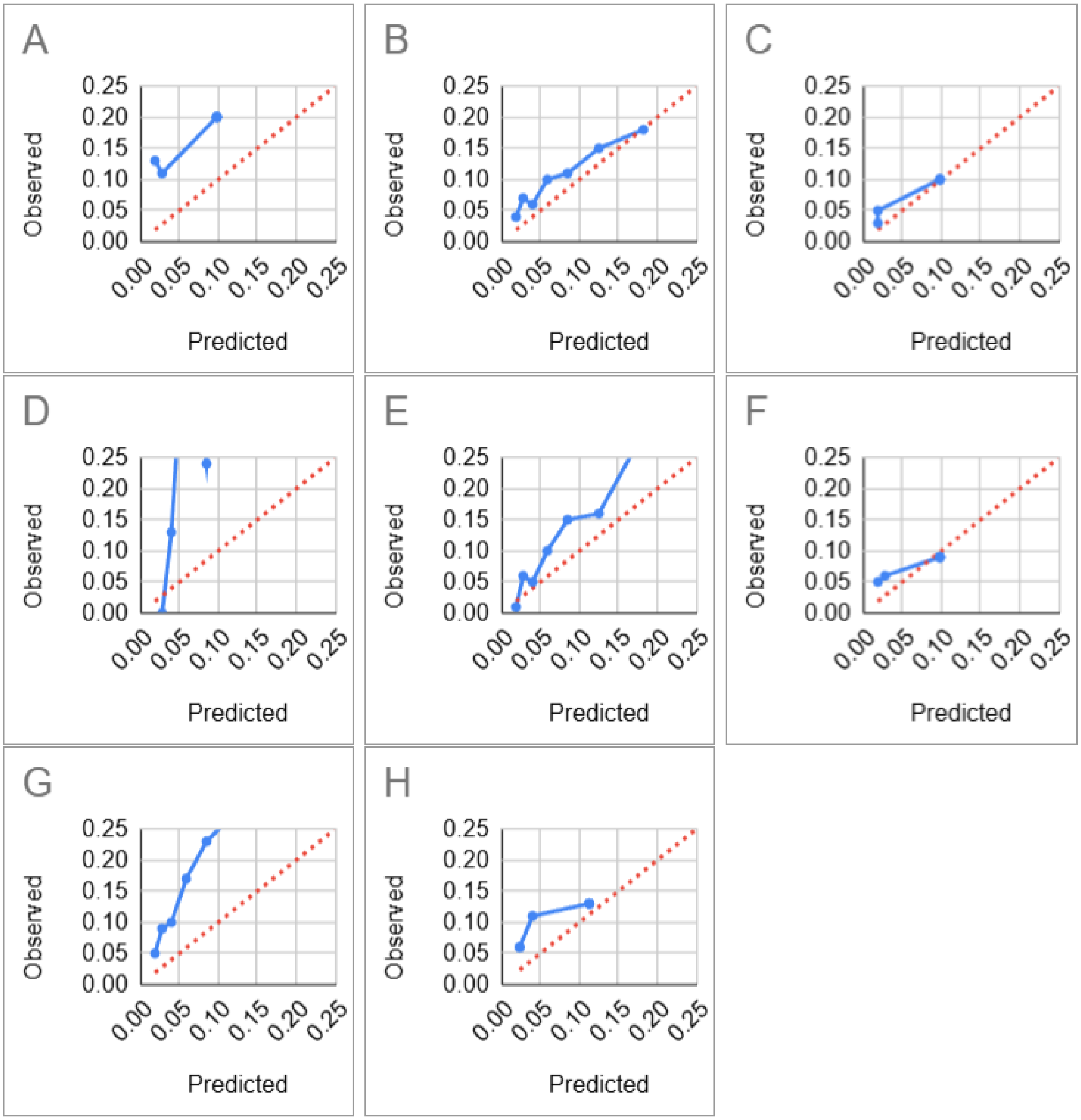


A. Hasegawa 2016
B. Shih 2016
C. Sood 2013
D. Wakasugi 2014
E. Wizeman 2010
F. Shah 2014
G. Chao 2014
H. Wetmore 2013

**Figure S9: Calibration plots for the HAS-BLED in all dialysis types**

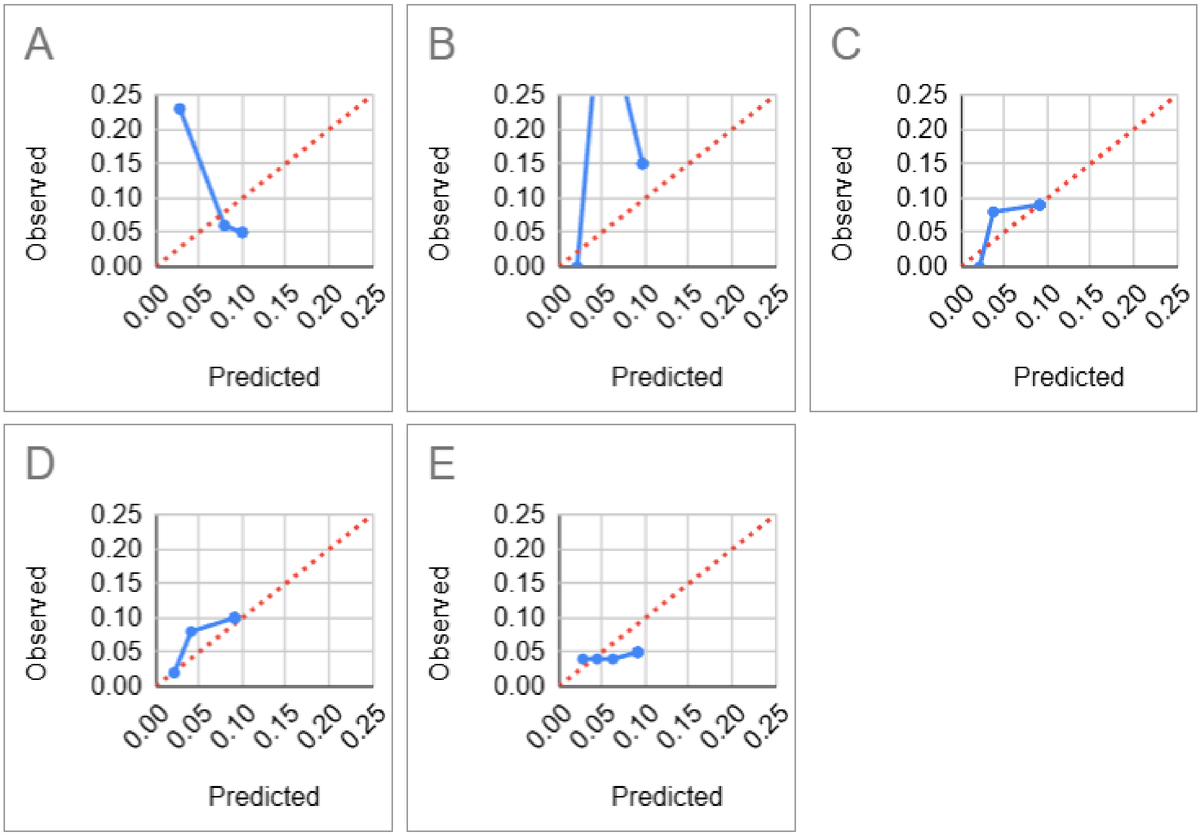


A. Bel-Ange 2021
B. Genovesi 2017
C. Shah 2014
D. Ocak 2019
E. See 2021

**SECTION D: RISK OF BIAS**

**Table S9: PROBAST signaling question scoring per study***

| Study/sign question | 1.1 | 1.2 | 2.1 | 2.2 | 2.3 | 3.1 | 3.2 | 3.3 | 3.4 | 3.5 | 3.6 | 4.1 | 4.2 | 4.3 | 4.4 | 4.5 | 4.6 | 4.7 | 4.8 | 4.9 |
|---|---|---|---|---|---|---|---|---|---|---|---|---|---|---|---|---|---|---|---|---|
| **Apostolakis**[37] | Y | Y | N | Y | Y | PN | PN | Y | N | Y | PN | N | Y | Y | NI | N.A. | NI | Y | N.A. | N.A. |
| **Bautista**[36] | PN | PN | Y | NI | Y | PN | NI | Y | NI | N | Y | Y | Y | Y | NI | N.A. | NI | Y | N.A. | N.A. |
| **Bel-Ange**[41] | Y | PY | Y | Y | Y | PN | Y | Y | N | NI | Y | Y | Y | Y | NI | N.A. | Y | Y | N.A. | N.A. |
| **Chan**[53] | PN | PY | N | NI | Y | N | NI | Y | N | NI | Y | N | Y | Y | PN | N.A. | Y | PN | N.A. | N.A. |
| **Chan**[34] | PN | N | Y | NI | Y | PN | Y | Y | N | NI | Y | N | Y | Y | NI | N.A. | Y | N | N.A. | N.A. |
| **Chao**[35] | PN | N | Y | NI | Y | PN | NI | Y | N | NI | PN | Y | Y | Y | NI | N.A. | NI | PY | N.A. | N.A. |
| **De Jong**[23] | PY | PN | Y | Y | Y | NI | Y | Y | NI | NI | Y | PY | Y | N | Y | N.A. | Y | Y | N.A. | N.A. |
| **De Jong**[5] | PN | PY | N | NI | Y | NI | PN | Y | NI | NI | Y | PY | Y | Y | N | N.A. | Y | Y | N.A. | N.A. |
| **Eikelboom**[67] | Y | Y | PN | Y | Y | PN | Y | Y | N | Y | Y | N | Y | Y | NI | N.A. | NI | N | N.A. | N.A. |
| **Friberg**[39] | PN | Y | Y | NI | Y | NI | NI | Y | NI | NI | PY | Y | N | Y | NI | N.A. | Y | PN | N.A. | N.A. |
| **Genovesi**[77] | Y | Y | Y | Y | Y | PN | NI | Y | NI | NI | Y | Y | Y | N | NI | N.A. | Y | N | N.A. | N.A. |
| **Hart**[68] | Y | N | Y | Y | Y | PN | Y | Y | N | N | Y | N | N | N | NI | N.A. | Y | N | N.A. | N.A. |
| **Hasegawa**[43] | PY | NI | N | Y | Y | NI | PN | Y | N | NI | Y | NI | Y | Y | NI | N.A. | NI | N | N.A. | N.A. |
| **Lee**[48] | PY | PN | Y | NI | Y | NI | Y | Y | Y | NI | Y | N | N | Y | NI | N.A. | Y | N | N.A. | N.A. |
| **McAlister**[72] | PN | N | N | NI | Y | NI | PN | Y | NI | NI | Y | Y | Y | NI | PY | N.A. | NI | Y | N.A. | N.A. |
| **Nakagawa**[73] | PN | Y | Y | NI | Y | PN | Y | Y | N | NI | Y | N | N | Y | NI | N.A. | Y | N | N.A. | N.A. |
| **Ocak**[3] | Y | Y | NI | Y | Y | NI | Y | Y | N | NI | Y | Y | PN | Y | Y | N.A. | Y | Y | N.A. | N.A. |
| **Schamroth Pravda**[52] | PN | Y | Y | NI | Y | NI | N | Y | NI | PN | Y | N | Y | Y | NI | N.A. | Y | PN | N.A. | N.A. |
| **Roldan**[40] | Y | PN | Y | Y | Y | NI | NI | Y | NI | Y | Y | N | Y | Y | NI | N.A. | NI | PN | N.A. | N.A. |
| **See**[50] | N | PN | NI | NI | Y | NI | PN | Y | NI | PN | Y | Y | N | N | NI | N.A. | N | N | N.A. | N.A. |
| **Shah**[49] | PN | N | Y | NI | N | NI | PN | Y | NI | NI | NI | Y | Y | Y | NI | N.A. | Y | N | N.A. | N.A. |
| **Shih**[44] | N | Y | Y | NI | Y | NI | Y | Y | NI | NI | Y | Y | N | Y | NI | N.A. | Y | N | N.A. | N.A. |
| **Sood**[45] | N | NI | N | NI | Y | NI | PN | Y | NI | NI | Y | N | Y | Y | NI | N.A. | Y | N | N.A. | N.A. |
| **Suzuki**[38] | Y | N | Y | Y | Y | Y | Y | Y | Y | NI | Y | Y | Y | Y | Y | N.A. | PY | PN | N.A. | N.A. |
| **Wakasugi**[46] | Y | N | N | Y | Y | PN | Y | Y | N | NI | NI | N | Y | Y | Y | N.A. | Y | N | N.A. | N.A. |
| **Wang**[33] | N | Y | Y | NI | Y | PN | Y | Y | N | NI | Y | Y | Y | Y | NI | N.A. | NI | PN | N.A. | N.A. |
| **Wetmore**[54] | PN | N | NI | NI | Y | NI | NI | NI | NI | NI | N | N | Y | Y | N | N.A. | Y | N | N.A. | N.A. |
| **Wizemann**[47] | N | NI | N | NI | Y | NI | PN | Y | NI | NI | NI | N | Y | Y | NI | N.A. | Y | N | N.A. | N.A. |
| **Xu**[82] | PN | Y | Y | NI | Y | PN | N | Y | N | NI | NI | N | Y | Y | NI | N.A. | Y | N | N.A. | N.A. |

| | | | | | | | | | | | | | | | | | | | | |
|---|---|---|---|---|---|---|---|---|---|---|---|---|---|---|---|---|---|---|---|---|
| **Akbar**[75] | N | Y | Y | NI | Y | NI | N | Y | NI | NI | N | N | Y | N | N | N.A. | Y | PN | N.A. | N.A. |
| **Barashi**[64] | N | PN | Y | NI | Y | PN | N | Y | PY | NI | PY | Y | Y | Y | Y | N.A. | PN | N | N.A. | N.A. |
| **Barcia**[65] | N | PY | Y | NI | Y | Y | N | Y | PN | NI | Y | Y | Y | NI | NI | N.A. | NI | N | N.A. | N.A. |
| **Calderon**[66] | N | PN | Y | NI | Y | NI | Y | Y | PY | NI | PY | Y | PY | Y | Y | N.A. | NI | N | N.A. | N.A. |
| **Elfar**[76] | N | PN | NI | NI | Y | NI | PN | Y | NI | NI | N | Y | Y | Y | PY | N.A. | NI | N | N.A. | N.A. |
| **Jun**[69] | PN | Y | Y | NI | Y | NI | PN | Y | PN | PY | PN | Y | PY | N | Y | N.A. | NI | N | N.A. | N.A. |
| **Kee**[70] | N | PN | NI | NI | PN | NI | PN | Y | PN | NI | PY | Y | PY | Y | Y | N.A. | Y | N | N.A. | N.A. |
| **Kim**[78] | N | PN | PY | NI | Y | NI | PN | Y | PY | NI | Y | N | Y | Y | Y | N.A. | Y | N | N.A. | N.A. |
| **Li**[71] | Y | Y | Y | Y | Y | NI | Y | Y | Y | Y | Y | Y | PY | Y | Y | N.A. | Y | N | N.A. | N.A. |
| **Ocak**[31] | Y | PN | Y | Y | Y | NI | Y | Y | PY | PY | Y | Y | PY | Y | Y | N.A. | Y | N | N.A. | N.A. |
| **Park**[83] | Y | Y | Y | Y | Y | Y | N | Y | NI | Y | Y | Y | Y | NI | NI | N.A. | NI | N | N.A. | N.A. |
| **Pokorney**[79] | Y | Y | Y | Y | Y | Y | Y | Y | PN | Y | PY | N | Y | Y | PY | N.A. | NI | N | N.A. | N.A. |
| **Reinecke**[80] | Y | PY | NI | Y | Y | NI | N | Y | NI | NI | Y | N | N | Y | NI | N.A. | NI | N | N.A. | N.A. |
| **Rivera-Caravaca**[74] | Y | Y | NI | Y | Y | NI | N | Y | NI | NI | Y | N | Y | Y | Y | N.A. | NI | N | N.A. | N.A. |
| **Sy**[51] | N | PN | PY | NI | N | NI | N | Y | NI | PN | NI | Y | Y | NI | NI | N.A. | Y | N | N.A. | N.A. |
| **Welander**[80] | PN | Y | Y | NI | Y | Y | Y | Y | PN | PN | NI | Y | Y | Y | NI | N.A. | Y | N | N.A. | N.A. |
| **Welander**[84] | N | PN | Y | NI | Y | Y | Y | Y | PN | PN | Y | PY | Y | Y | NI | N.A. | Y | N | N.A. | N.A. |
| **Wetmore**[85] | N | PY | Y | NI | Y | Y | Y | Y | PN | PN | NI | Y | PY | Y | NI | N.A. | Y | N | N.A. | N.A. |

* Y = yes, PY = probably yes, NI = no information, PN = probably no, N = no, N.A. = not applicable

**Table S10: PROBAST Risk of Bias (ROB) per study***

| Risk of Bias | Domain | | | | |
|---|---|---|---|---|---|
| **Study** | **Participants** | **Predictors** | **Outcome** | **Analysis** | **Total** |
| **Apostolakis[37]** | + | - | - | - | - |
| **Bautista[36]** | - | ? | - | ? | - |
| **Bel-Ange[41]** | + | + | - | ? | - |
| **Chan[53]** | - | - | - | - | - |
| **Chan[34]** | - | ? | - | - | - |
| **Chao[35]** | - | ? | - | ? | - |
| **De Jong[23]** | - | + | ? | - | - |
| **De Jong[5]** | - | - | - | - | - |
| **Eikelboom[67]** | + | - | - | - | - |
| **Friberg[39]** | - | ? | ? | - | - |
| **Genovesi[77]** | + | + | - | - | - |
| **Hart[68]** | - | + | - | - | - |
| **Hasegawa[43]** | ? | - | - | - | - |
| **Lee[48]** | - | ? | ? | - | - |
| **McAlister[72]** | - | - | - | ? | - |
| **Nakagawa[73]** | - | ? | - | - | - |
| **Ocak[3]** | + | ? | - | - | - |
| **Schamroth Pravda[52]** | - | ? | - | - | - |
| **Roldan[40]** | - | + | ? | - | - |
| **See[50]** | - | ? | - | - | - |
| **Shah[49]** | - | - | - | - | - |
| **Shih[44]** | - | ? | ? | - | - |
| **Sood[45]** | - | - | - | - | - |
| **Suzuki[38]** | - | + | ? | - | - |
| **Wakasugi[46]** | - | - | - | - | - |
| **Wang[33]** | - | ? | - | - | - |
| **Wetmore[54]** | - | ? | - | - | - |
| **Wizemann[47]** | - | - | - | - | - |
| **Xu[82]** | - | ? | - | - | - |
| **Akbar[75]** | - | ? | - | - | - |
| **Barashi[64]** | - | ? | - | - | - |
| **Barcia[65]** | - | ? | - | - | - |
| **Calderon[66]** | - | ? | ? | - | - |
| **Elfar[76]** | - | ? | - | - | - |
| **Jun[69]** | - | ? | - | - | - |
| **Kee[70]** | - | - | - | - | - |
| **Kim[78]** | - | ? | - | - | - |
| **Li[71]** | + | + | ? | - | - |
| **Ocak[31]** | - | + | ? | - | - |
| **Park[83]** | + | + | - | - | - |
| **Pokorney[79]** | + | + | - | - | - |

| | | | | | |
|---|---|---|---|---|---|
| **Reinecke[80]** | + | ? | - | - | - |
| **Rivera-Caravaca[74]** | + | ? | - | - | - |
| **Sy[51]** | - | - | - | - | - |
| **Welander[80]** | - | ? | - | - | - |
| **Welander[84]** | - | ? | - | - | - |
| **Wetmore[85]** | - | ? | - | - | - |

* + indicates low ROB; - indicates high ROB; ? indicates unclear ROB. A domain was classified as low ROB if all signaling questions in that domain were answered as ‘yes’ or ‘probably yes’, as high ROB if at least one signaling question in that domain was answered as ‘no’ or ‘probably no’ and as unclear ROB if at least one signaling question was answered as ‘no information’ and the remaining signaling questions in that domain were answered as ‘yes’ or ‘probably yes’.

**Figure S10: Funnel plots**

Figure a contains the funnel plots for CKD only, figure b for dialysis only and figure c for CKD and dialysis combined. These funnel plots were created using the c-statistics of the main analysis, their 95% confidence intervals and their corresponding standard errors. Funnel plots of a prediction model are only presented if two or more studies presenting c-statistics were present, therefore figure a does not contain any bleeding models and figure b and c only contain the HAS-BLED.

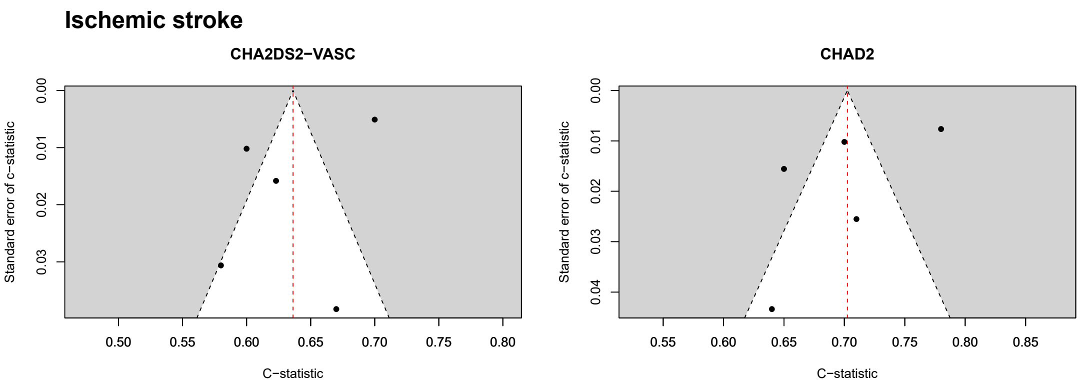


a)

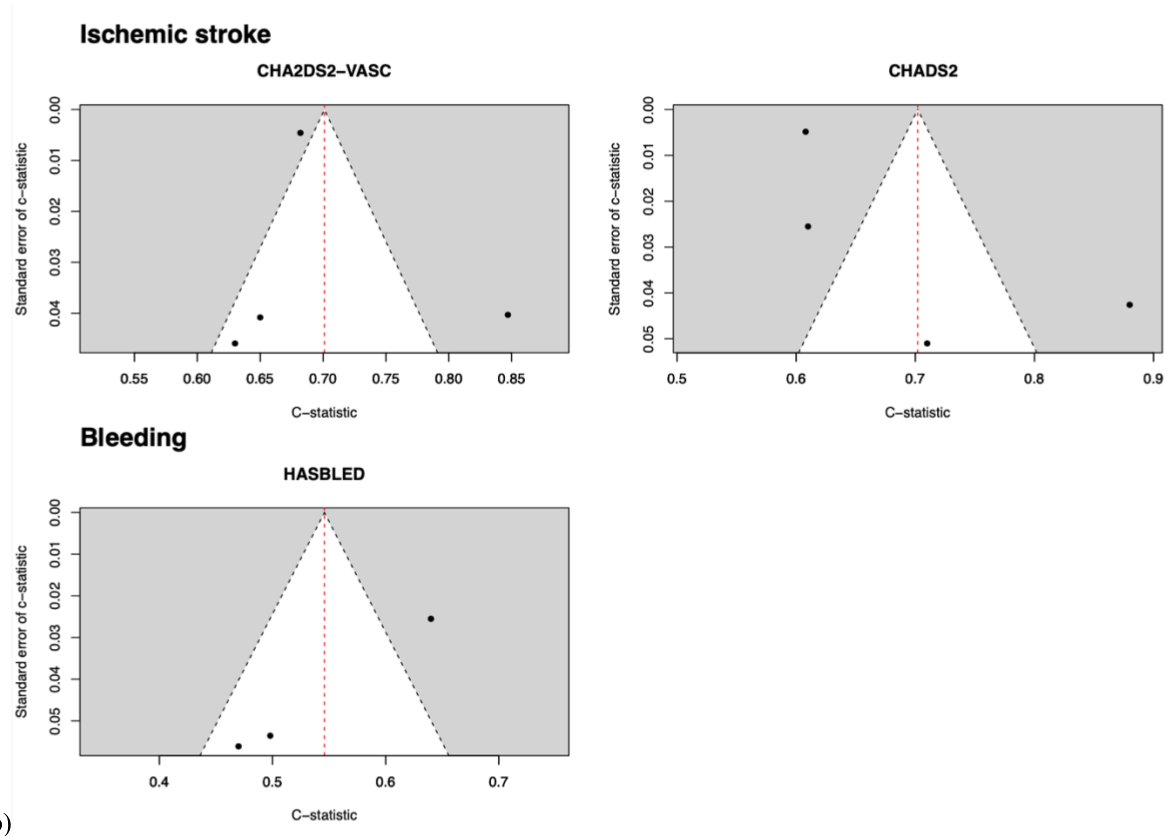


b)

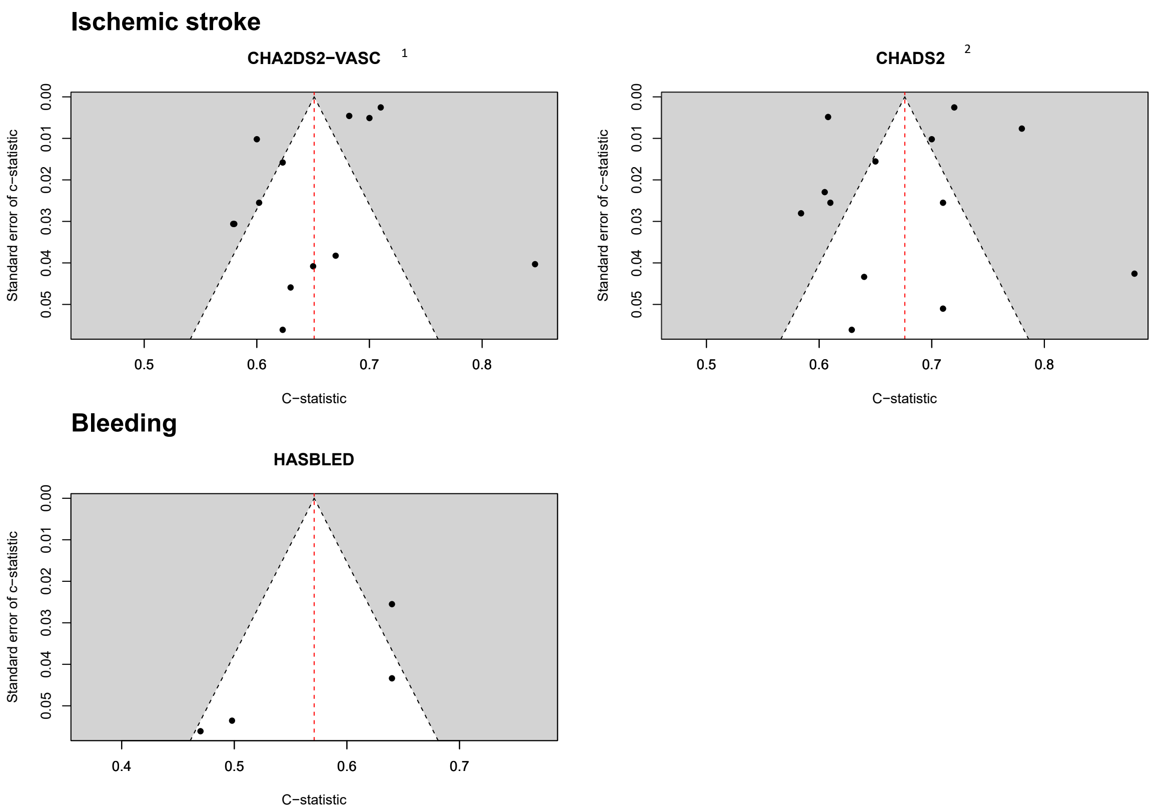


c)

1. Egger’s test for funnel plot asymmetry: p=0.61, 2. Egger’s test for funnel plot asymmetry: p=0.27
